# Acetylation regulates the oligomerization state and activity of RNase J, the *Helicobacter pylori* major ribonuclease

**DOI:** 10.1038/s41467-023-43825-8

**Published:** 2023-12-06

**Authors:** Alejandro Tejada-Arranz, Aleksei Lulla, Maxime Bouilloux-Lafont, Evelyne Turlin, Xue-Yuan Pei, Thibaut Douché, Mariette Matondo, Allison H. Williams, Bertrand Raynal, Ben F. Luisi, Hilde De Reuse

**Affiliations:** 1 Département de Microbiologie, Unité Pathogenèse de Helicobacter, UMR CNRS 6047, Institut Pasteur, Paris, France; 2 Université de Paris, Sorbonne Paris Cité, Paris, France; 3 Department of Biochemistry, University of Cambridge, Tennis Court Road, Cambridge, CB2 1GA UK; 4 Plateforme Protéomique, Unité de Spectrométrie de Masse pour la Biologie, C2RT, USR CNRS 2000, Institut Pasteur, Paris, France; 5 University of California San Francisco, Cellular Molecular Pharmacology, San Francisco, CA USA; 6 Département de Biologie structurale et chimie, Plateforme de biophysique moléculaire, Institut Pasteur, Paris, France; 7 Present Address: Biozentrum, University of Basel, Basel, Switzerland

**Keywords:** Acetylation, RNA decay

## Abstract

In the gastric pathogen *Helicobacter pylori*, post-transcriptional regulation relies strongly on the activity of the essential ribonuclease RNase J. Here, we elucidated the crystal and cryo-EM structures of RNase J and determined that it assembles into dimers and tetramers in vitro. We found that RNase J extracted from *H. pylori* is acetylated on multiple lysine residues. Alanine substitution of several of these residues impacts on *H. pylori* morphology, and thus on RNase J function in vivo. Mutations of Lysine 649 modulates RNase J oligomerization in vitro, which in turn influences ribonuclease activity in vitro. Our structural analyses of RNase J reveal loops that gate access to the active site and rationalizes how acetylation state of K649 can influence activity. We propose acetylation as a regulatory level controlling the activity of RNase J and its potential cooperation with other enzymes of RNA metabolism in *H. pylori*.

## Abstract (web summary)

Here the authors find that RNase J, the major ribonuclease of the gastric pathogen *Helicobacter pylori* is post-translationally modified by acetylation. They show that acetylation can control RNase J activity.

## Introduction

Post-transcriptional regulation is one of the most important levels of control of gene expression in every kingdom of life. Ribonucleases (RNases) are key enzymes in this regulation and are often involved in processes of both RNA maturation and degradation. In several bacteria, major RNases act in concert with DEAD-box RNA helicases, that help to unfold RNA secondary structures, within protein machines called RNA degradosomes^1^.

We previously demonstrated the existence of a minimal RNA degradosome in *Helicobacter pylori*^2^. This Gram-negative bacterial pathogen chronically colonizes the stomach of half of the human population worldwide. Infection leads to chronic gastritis and, in some cases, to further gastric pathologies such as peptic ulcers, MALT lymphoma, or gastric adenocarcinoma that causes 800,000 deaths each year worldwide^3^. *H. pylori* possesses a small genome (1.6 Mb) with few transcriptional regulators, and several lines of evidence indicate that post-transcriptional regulation plays a major role in gene expression control in this organism^4–6^. The *H. pylori* RNA degradosome is composed of RNase J, a 77.6 kDa enzyme that possesses both 5′−3′ exoribonuclease and endoribonuclease activities, and RhpA, a DEAD-box RNA helicase, with a molecular weight of 55.8 kDa^2,7^. RNase J is essential in *H. pylori* and plays a major role in the degradation of both mRNAs and antisense RNAs^8^. RhpA is the sole DEAD-box RNA helicase encoded by the *H. pylori* genome and is essential for colonization in the mouse infection model^7^. Furthermore, we recently demonstrated that, in *H. pylori*, the RNA degradosome is compartmentalized into large clusters at the inner membrane, that comprise up to 20 RNase J molecules, and whose formation is regulated and likely represents a major level of control of its activity^9^.

RNase J-encoding genes are present in 57% of sequenced bacteria^1^, including the Gram-positive organisms *Bacillus subtilis*, *Staphylococcus aureus* and *Deinococcus radiodurans*. *B. subtilis* and *S. aureus* encode two paralogues of RNase J, called J1 and J2 while *H. pylori, Streptomyces pyogenes* and *D. radiodurans* possess only one gene for this protein. Compared to the RNase J proteins of these three organisms, the *H. pylori* enzyme possesses a unique N-terminal extension of 132 residues that is predicted to be intrinsically disordered^2,9^. In *B. subtilis*, both RNase J paralogues (*Bsu*RNases J1 and J2) have equivalent endoribonuclease activities, but *Bsu*RNase J2 seems to lack the 5’−3’ exoribonuclease activity that predominates in *Bsu*RNase J1^10^. Furthermore, these two proteins interact and form a hetero-tetramer in vitro that has different cleavage specificity in vitro than each protein alone^10^. In vitro analysis of the RNase J1 enzyme from *S. aureus* showed that it assembles mostly into dimers, but also forms tetramers, independently of divalent cations^11^. The singly encoded RNase J from *S. pyogenes* forms dimers and tetramers in vitro without divalent cations being observed in the dimerization surface^12^. In *D. radiodurans*, RNase J forms a homodimer in a Mn-dependent fashion in vitro, and the interaction between the protomers is mediated by the C-terminal domain (CTD) of *Dra*RNase J^13^. Several crystal structures of RNase J proteins have been reported^12–14^, generally revealing tetrameric quaternary structures (composed of dimers of dimers). Whether the different oligomeric states of RNase J detected in vitro have differences in activity has not been explored.

Another factor that could in principle influence activity of RNase J is post-translational modification. Acetylation has emerged as a major post-translational regulatory mechanism in bacteria^15,16^. Although the acetylation of lysine, cysteine, serine and threonine residues has been reported, acetylation of the lysine ε-amino group is the most prominent modification^15,17^. This modification has been shown to regulate multiple properties of the target proteins, including enzymatic activity, DNA-binding capability or stability, among others^16^. Interestingly, some proteins involved in RNA metabolism have been reported to be lysine acetylated, including (i) *Escherichia coli* RNase R, whose acetylation regulates its stability^18^; (ii) *E. coli* RNase II, regulated at the activity level^19^; and (iii) the CshA DEAD-box RNA helicase from *B. subtilis*, where acetylation has been proposed to influence its interaction with RNA polymerase independently from its association with RNase Y, the major ribonuclease of the *B. subtilis* RNA degradosome^20^.

The central importance of RNase J in post-transcriptional regulation in *H. pylori* and its participation in an RNase J-based degradosome prompted us to investigate the structure, oligomeric state and post-translational regulation of the *H. pylori* RNase J. Here, we report the crystal and cryo-EM structures of *H. pylori* RNase J that assembles in tetramers organized as a dimer-of-dimers. We also show that in vitro and in solution, RNase J is in equilibrium between dimeric and tetrameric states and our data suggest that the tetrameric form constitutes the active form of RNase J in vitro. Furthermore, we found that in *H. pylori* RNase J is acetylated at several residues by multiple mechanisms and that mutations of several of these acetylated residues influence RNase J activity in *H. pylori*. We concluded that the acetylation state regulates the activity of RNase J in vitro and most probably in *H. pylori*, and thereby constitutes an important level of control of RNA metabolism.

## Results

### Structure of the *H. pylori* RNase J

We previously reported that the N-terminal part of RNase J (first 120 amino acids) that is specific of the *Helicobacter* species is predicted to be an intrinsically disordered region^9^. Using the tool Robetta, based on machine learning^21^, this region is predicted with low confidence to have an extended helical region of the N-terminal residues 1–136, and this region seems unlikely to have a compact fold.

**Fig. 1:**
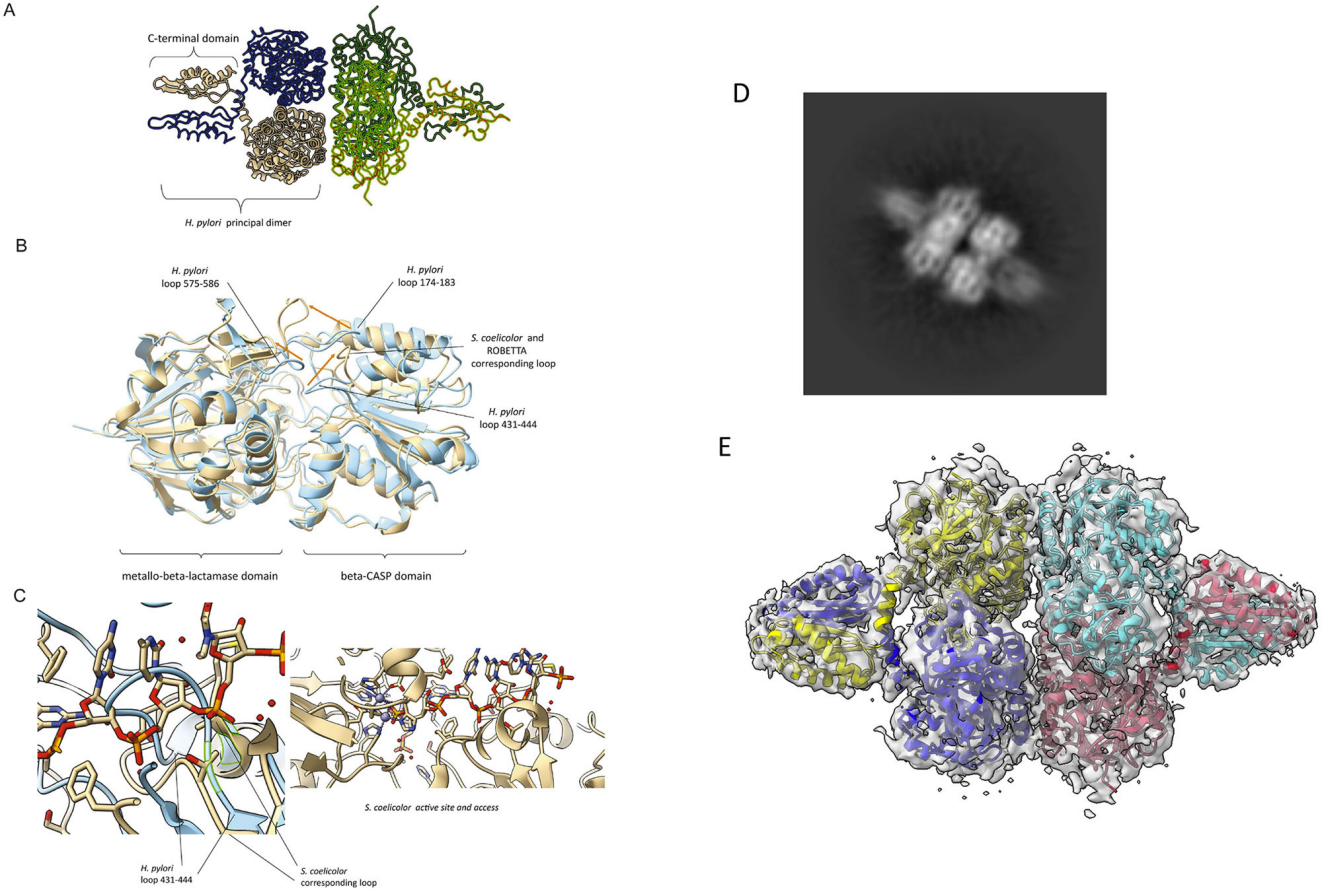
Structure of RNase J from *H. pylori*. **A** Crystal structure of RNase J. View of the tetramer, which is a dimer-of-dimers. A monomer occupies the asymmetric unit, and the tetramer is generated through crystallographic symmetry. Crystals were prepared from *H. pylori* RNase J residues 139–691, encompassing the beta-CASP, metallo-beta-lactamase and C-terminal domains. **B** Overlay of the predicted structure from Robetta server (beige) and the refined crystal structure (blue). The individual beta-CASP and metallo-beta-lactamase domains overlay well, but there are noted differences in the orientation of the three loop regions 174–183, 431–441 and 575–586 at the entrance to the active site (shifts indicated by orange arrows). The predicted structure is closer to the orientation seen in *Streptomyces coelicolor* RNase J, shown in overlay with *H. pylori* enzyme in the left panel of (**C**). The *S. coelicolor* enzyme structure (beige) is in complex with RNA, and the path of the RNA substrate along a channel to the active site is shown in the right panel. The *H. pylori* RNase J (blue) cannot bind RNA with the same orientation seen for the *S. coelicolor* enzyme due to steric clashes with the loops (left panel). The two zinc ions at the active site of the *S. coelicolor* enzyme are indicated in the right panel as blue spheres. **D** 2D class average from cryo-EM analysis of RNase J. **E** The 3D cryo-EM map with fitted model based on the crystal structure of the dimer and predicted model for the C-terminal dimerization domain. The RNase J protomers of one dimer are colored yellow and blue, and for the second dimer are red and cyan. The cryoEM density map is in gray.

Using an RNase J protein truncated of this region (comprising residues 137–691 from *H. pylori* strain 26695, Uniprot P56185) we were able to obtain well-diffracting crystals. The crystal structure was solved at 2.75 Å by molecular replacement using the *Streptomyces coelicolor* homolog as a search model (Supplementary Table 1A). One molecule occupies the asymmetric unit of the crystal, and through crystal symmetry a homotetramer can be generated that is organized as a dimer-of-dimers (Fig. 1A). The core region of the enzyme, residues 137–586, encompasses the beta-CASP and beta-lactamase domains that define this family^22^, but there are no detectable metal cofactors in the map at the catalytic site. An X-ray fluorescent scan also confirmed that there are no detectable Zn ions in the crystal. The CTD, from residues 587 to 691, is poorly ordered in the crystal lattice and the density was not sufficiently well resolved to model the polypeptide.

The structure predicted by the Robetta server is in excellent agreement with the X-ray model for the core, with the notable exception of the three loop regions 174–183, 431–441 and 575–586 at the entrance to the active site, for which the predicted loop positions are closer to those seen in the crystal structure of the *S. coelicolor* RNase J (Fig. 1B, C). The electron density over these regions is well defined in the *H. pylori* map and modeled with high confidence.

A cryo-EM structure was obtained from purified full-length RNase J protein (Fig. 1D, E and Supplementary Table 1B). The enzyme has a preferred orientation in the ice, and this has limited the resolution of the reconstructions to 4.1 angstroms (Supplementary Fig. 1). However, the reconstructions reveal unequivocally a tetrameric quaternary structure and resolve the core as well as the C-terminal oligomerization domain. The Robetta model for the CTD fits well into the map, showing good agreement with the predicted helical segments. The predicted model is also in agreement with a high confidence homology model prepared by PHYRE2 using the *Thermus thermophilus* homolog as template (PDB 3BK2).

In the dimeric unit, the protomer interfaces are formed by self-complementary interactions made mostly by the CTD. The surface area buried at the CTD–CTD interface is estimated to be 1165 Å^2^. The beta-CASP and beta-lactamase domains of RNase J also make a self-complementary interface in the principal dimer, with an estimated area of 672 Å^2^ that is a little more than half of the CTD-CTD contribution. Stronger interactions typically have a surface area of more than 2000 Å^2 ^^23^. The interface between the beta-CASP and beta-lactamase domains is very similar to that seen in other dimers of RNase J, for instance in the homologue of *S. coelicolor*^12^. The interfaces that are formed between the dimer units to make the tetramer are also made by the beta-CASP and beta-lactamase interfaces, and these have a total buried area of 2070 Å^2^.

An overlay of RNase J of *H. pylori* and of *S. coelicolor*, where an RNA substrate is bound, suggests that the path of the RNA to the active site cannot be the same for the two enzymes, and that the nucleic acid would need to be rerouted in the *H. pylori* enzyme (Fig. 1C). The obstructions are caused by exposed peptide loop regions that are positioned to accommodate the CTD. It is possible that changes in the oligomerization state will impact on the loops and access to the active site.

### In vitro analysis of the oligomeric state of the RNase J and RhpA proteins

**Fig. 2:**
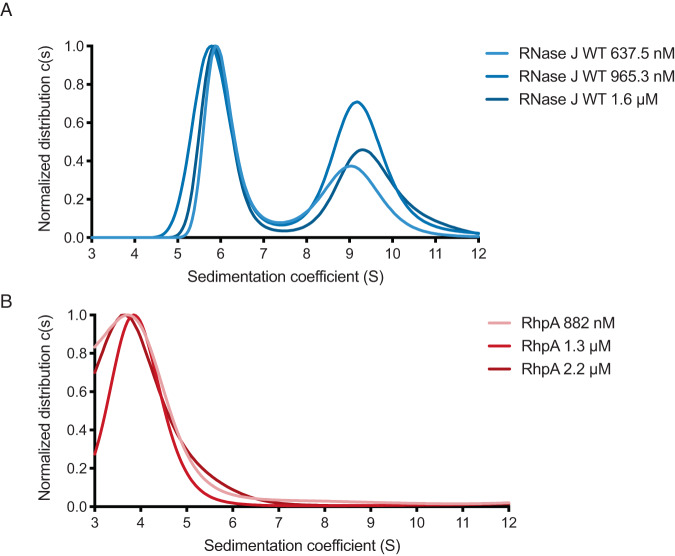
The solution oligomerization state of RNase J and RhpA proteins. The profiles are from analytical ultracentrifugation sedimentation of (**A**) His-tagged RNase J and (**B**) His-tagged RhpA. The profiles were recorded at 230 nm wavelength and normalized to 1 for the highest peak of each analysis. Source data are provided as a Source Data file.

The structure of *H. pylori* RNase J and of several other RNase J proteins correspond to tetramers. However, for some of these proteins, other oligomeric states (dimers and monomers) were found in solution and the activity of the different forms was unclear. To evaluate the oligomeric state of *H. pylori* RNase J and RhpA in vitro, we performed analytical ultracentrifugation (AUC) with His-tagged versions of RNase J and RhpA purified from *E. coli*. The sedimentation coefficient distribution of RNase J indicates that this protein forms two main oligomeric species in vitro with concentration-independent sedimentation coefficients (S_0_) of 5.8 and 8.8 (Fig. 2A). Based on the structural data of *H. pylori* RNase J presented above, these two species correspond to a dimer and a tetramer (Fig. 2A) that have a structural arrangement in solution which is compatible with the observed crystal structure. In mass photometry experiments, the major form of RNase J is a tetramer (Supplementary Fig. 2A). AUC of RhpA corresponds to a dimer independently of its concentration with a sedimentation coefficient of 4.5 (Fig. 2B), a result that is consistent with mass photometry (Supplementary Fig. 2B).

Our attempts to reliably identify a stable RNase J-RhpA complex in vitro by gel filtration, AUC and mass photometry were unsuccessful (Supplementary Fig. 2C). Although RNase J and RhpA were copurified in vivo from both *H. pylori* and *E. coli*, these in vitro data suggest that these two proteins form a complex of a transient nature that may be facilitated by the presence of RNA or rely on condensate-like environments and association with the membrane, as we previously reported in *H. pylori*^9^.

### Bacterial two hybrid (BACTH) to identify residues important for the oligomerization of RNase J

**Fig. 3:**
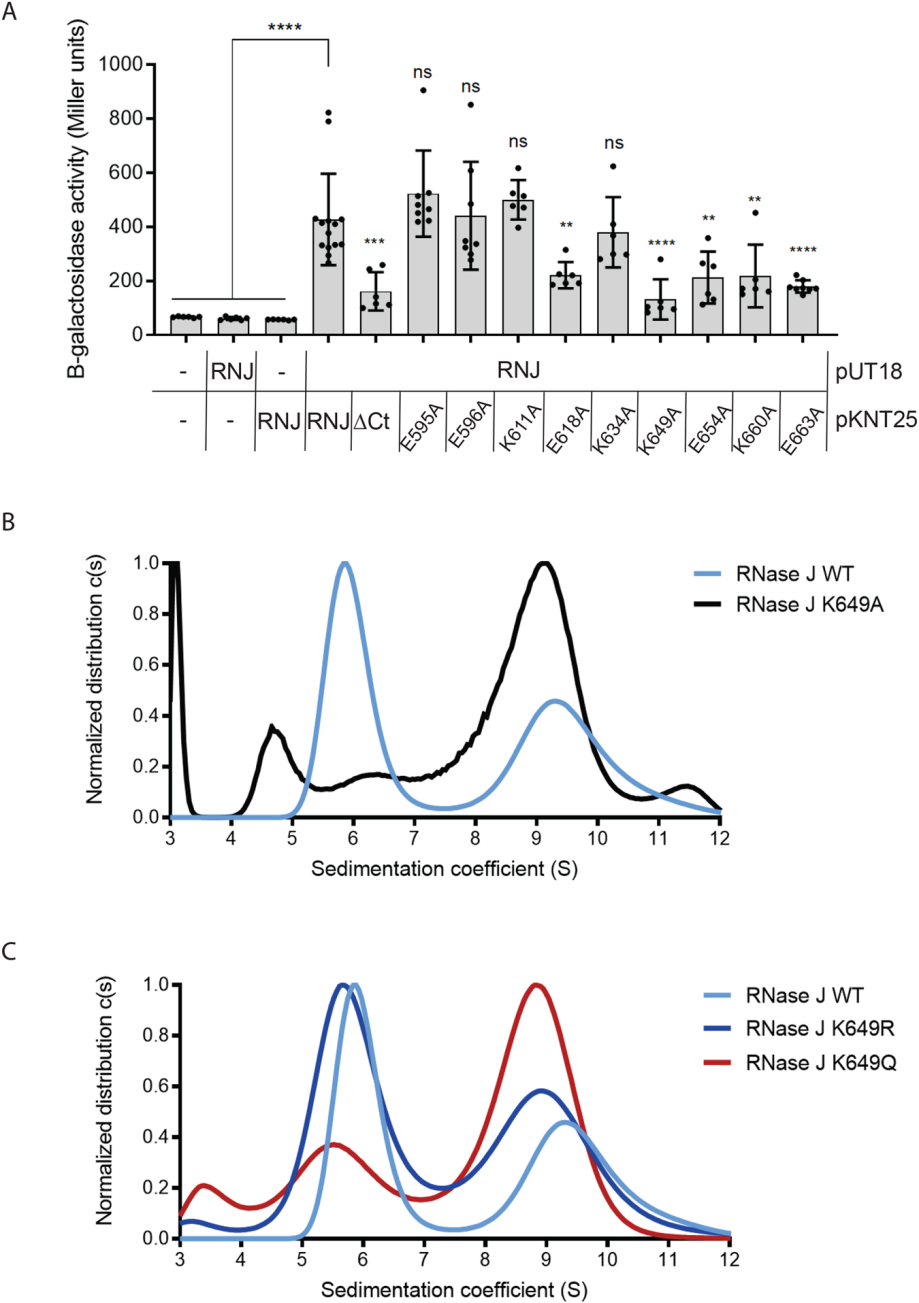
Point mutations modify the oligomeric state of RNase J. **A** BACTH two hybrid experiments to assess homo-oligomerization of WT and mutant RNase J proteins. Beta-galactosidase activity data (*n* ≥ 6) are presented as bar graphs of mean values with SD, individual measurements are indicated. Differences in the beta-galactosidase activity units are indicated with ns (non-significant) **p-value < 0.005, ***p-value < 0.0005, ****p-value < 0.0001. The statistic test applied was one-way ANOVA with Dunnett’s multiple comparisons test. **B** Analytical ultracentrifugation sedimentation profiles of His-tagged RNase J WT and variant K649A at 230 nm wavelength. **C** Analytical ultracentrifugation sedimentation profiles of RNase J WT and variants K649R and K649Q at 230 nm wavelength. Source data are provided as a Source Data file.

We then searched for residues potentially involved in the self-interaction of RNase J using bacterial two hybrid (BACTH) experiments that test the interaction of protein pairs in *E. coli*. First, a consistent interaction of wild type RNase J with itself was detected (Fig. 3A). As previously reported for other RNase J proteins^13^, we also found that the C-terminal region of RNase J is essential for this interaction. In order to pinpoint residues that are important for RNase J multimerization, we constructed RNase J mutants in which nine charged residues of this CTD were individually replaced by alanine residues. As shown in Fig. 3A, several mutants were significantly affected in their capacity to homo-oligomerize, including RNase J E618A, K649A, E654A, K660A and E663A. These data show that charged residues at the CTD of RNase J are important for its self-interaction.

### RNase J is acetylated at several lysine residues

In *E. coli*, RNase R and RNase II have been reported to be post-translationally modified by acetylation but no such modification was reported on RNase J. A mass spectrometry (MS) analysis was performed on an RNase J-FLAG fusion protein expressed from the *H. pylori* chromosome under the control of its native promoter. The RNase J-FLAG protein was pulled down from *H. pylori* crude lysates using anti-FLAG antibodies and extracted from a polyacrylamide gel. The experiment was performed on three biological replicates from cultures in exponential phase. We found that nine lysine residues of RNase J, spread across all protein domains, are consistently acetylated: K134, K140, K323, K337, K397, K511, K547, K634 and K649 (Supplementary Fig. 3 and Supplementary data 1).

### Lysine 649 is important for the oligomerization state of RNase J

Lysine 649 (K649), localized at the RNase J CTD, was particularly interesting since this residue was both acetylated and required for RNase J homo-oligomerization in two hybrid experiments. Therefore, AUC analyses were performed with purified wild type RNase J and variants (Supplementary Fig. 4) to test the importance of K649 in RNase J homo-oligomerization. As shown in Fig. 3B, the purified RNase J K649A mutant assembles exclusively as a tetramer in vitro, with a complete loss of the dimeric population seen with the WT protein. Circular dichroism was performed to verify that this effect was not due to a defect in the folding of RNase J K649A. The circular dichroism spectra of wild-type and K649A variants of RNase J did not show significant differences (Supplementary Fig. 5) and the estimated proportions of the different secondary structure elements were maintained (Supplementary Table 2). This shows that mutation K649A does not introduce a major folding defect in RNase J secondary structure. Thus, K649 is important for RNase J oligomerization both in vitro and in *E. coli*.

### Acetylation mimicry of Lysine 649 affects the oligomeric state of RNase J

Given that K649 is acetylated, we next investigated whether RNase J acetylation at this residue could represent a control level of its oligomeric state. Therefore, we constructed RNase J mutants in which K649 was either replaced by an arginine residue (K649R), that mimics a non-acetylated state but maintains a positive charge at this position, or replaced by a glutamine residue (K649Q) which mimics an acetylated state while neutralizing the positive charge [a previously validated strategy^24^]. As shown in Fig. 3C, the AUC sedimentation profile of RNase J K649R is similar to that of the wild type enzyme. In contrast, RNase J K649Q shows a significantly increased proportion of tetramers over the dimers, similarly to the K649A mutant (Fig. 3B, C). These data show that the positive charge of K649 is important for the stability of the RNase J dimeric state and that oligomerization can be modulated by the charge state of K649.

### Mutations in K649 alter the exoribonuclease activity of RNase J

**Fig. 4:**
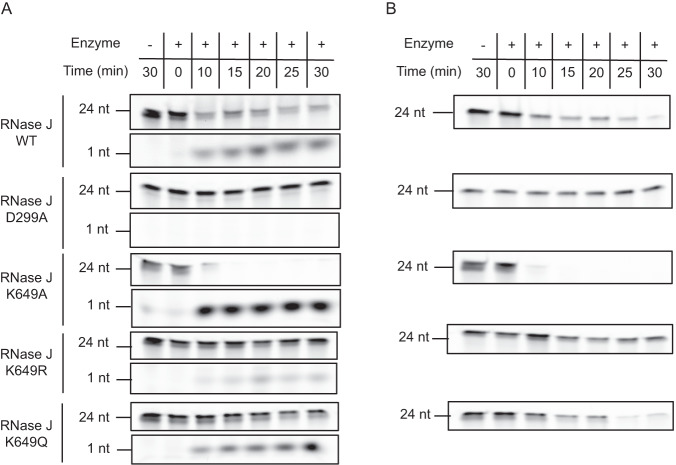
Exoribonuclease activity of RNase J wild type and mutants. **A** Exoribonuclease activity of His-tagged RNase J WT and different variants using a 24-nt RNA substrate labeled with 6-carboxyfluorescein (6-FAM) at its 5’-end over the course of 30 min. **B** Exoribonuclease activity of RNase J WT and different variants using a 24-nt RNA substrate labeled with 6-FAM at its 3’-end over the course of 30 min. Experiments were performed in independent triplicates. Source data are provided as a Source Data file.

Since RNase J WT forms both dimers and tetramers in vitro, we next assessed whether the oligomeric state and the presence of a charge at position 649 were correlated with differences in the in vitro activity of this protein. The exoribonuclease activity of His-tagged RNase J was measured with a 24-nt long single stranded RNA substrate from the 5’-untranslated region (UTR) of *rnj*, upon which we previously found RNase J to be active^8^. This substrate was labeled with 6-carboxyfluorescein (6FAM) at either the 3’ or 5’-ends. As a control, a mutant inactivated at the RNase J active site, D299A, was used. We found, as expected, that the D299A inactive mutant did not degrade the substrate while the wild type RNase J was active in its 5’−3’ exoribonucleolytic mode (Fig. 4A, B and Table 1). This activity is evident from the rapid depletion in the intact substrate bands labeled in 5’ (Figs. 4A) or 3’ (Fig. 4B) and, from the additional accumulation of the 1 nt product upon digestion of the 5’-labeled substrate (Fig. 4A).

**Table 1:** Half-lives of fluorescent RNA substrates in the presence of different variants of RNase J alone or in complex with RhpA

	Half-life $\pm$ SD (min)			
RNase J variant	5’-labeled substrate (exo)	3’-labeled substrate (exo)	3’-labeled substrate with hairpins (endo)	3’-labeled substrate with hairpins + RhpA (complex)
WT	18.77 ± 3.16	7.37 ± 3.36	31.36 ± 17.57	6.29 ± 2.92
D299A	>30 [165.04 ± 93.36]	>>>30	>75 [231.05 ± 163.38]	n.d.
K649A	4.52 ± 0.52	2.75 ± 0.82	10.3 ± 0.56	6.95 ± 0.49
K649R	>30 [222.80 ± 175.05]	>30 [36.61 ± 12.04]	>75 [81.83 ± 6.81]	27.70 ± 5.4
K649Q	>30 [56.80 ± 13.02]	14.82 ± 9.11	>75 [92.52 ± 26.85]	>30 [37.33 ± 28.89]

The half-live values in brackets are estimated values extrapolated from the decay slope when the half-lives exceeded the time of the assay, over 30 min for the two first and the last columns and over 75 min for the third column. Experiments were performed in independent triplicates. Source data are provided as a Source Data file.

The RNase J mutant with K649 replaced by R, that mimics a non-acetylated state, and which behaves as dimers and tetramers like the WT protein, was strongly affected in its exoribonuclease activity (Fig. 4 and Table 1). For the K649R variant, the half-lives of the 3’- and 5’-labeled substrates increased by 5 and 10-fold, respectively (Table 1).

In contrast, the protein variants with K649 replaced either by A (that abolishes both the positive charge at this position and the acetylation) or by Q (acetylation-mimicking mutation) and that both mainly assembled into tetramers, presented an activity that was significantly higher than that of the RNase J K649R variant. The half-lives of 3’ and 5’-labeled substrates were 13 and 49-fold lower for the K649A variant and 2.5 and 4-fold lower for the K649Q variant, respectively. The half-lives of the substrates were calculated from three independent experiments for the wild type and for every RNase J variant and are shown in Table 1.

From these data, we conclude that, for these mutants, the tetrameric form of RNase J is most probably the active form in vitro. Altogether, we identified a residue, K649, whose charge is important for RNase J activity and oligomerization and that is acetylated in *H. pylori* cells. This suggests a mechanism of control of RNase J activity that is based on the allosteric control of its oligomerization by acetylation.

### Functional cooperation of RNase J-RhpA is not affected by K649 mutations

Our previous data have shown that RNase J-RhpA interact and cooperate to accelerate degradation of structured RNAs^8^. In this functional cooperation, RhpA could unwind the secondary structure elements and allow RNase J to act in its exoribonucleolytic mode. Here, using highly purified proteins, we were not able to detect a direct physical interaction between RNase J and RhpA in vitro, suggesting that their cooperation may not involve a stable interaction.

It is not known whether the oligomerization state of RNase J can affect the formation of transient interactions with the helicase or cooperation of the two enzymes. Therefore, we wanted to determine whether mutations of K649 affect the activity of the RNase J-RhpA mixture on structured RNAs. For this, we used a 45-nt long RNA substrate with hairpin structures at both ends to prevent the exoribonuclease activity from the 5’-extremity and labeled it with 6FAM at the 3’ end [as in^13^, Supplementary Fig. 6A]. First, the activity of RNase J WT and mutant proteins alone was tested on this substrate (Supplementary Fig. 6B). As compared to the linear 3’-labeled substrate, the degradation kinetics of this substrate by RNase J was significantly reduced but not abolished (Table 1), suggesting that RNase J might attack this substrate by its endoribonuclease activity and then proceeds by exoribonuclease activity. Most interestingly, the activity tests with a mixture of RNase J and RhpA in a 1:1 ratio resulted in a significant decrease in the half-life of the substrate to 6.3 ±2.9 min as compared to the activity of RNase J alone (approx. 31.4 ±17.6 min), compatible with a functional complex (Table 1). The half-life of the structured substrate incubated with the complex between RhpA and the RNase J K649A variant (that has increased exoribonuclease activity) was not affected (half-life 6.7 ±0.5 min) and significant activity of the RhpA-RNase J K649R complex was measured (half-life 27.7 ±5.4 min) (Supplementary Fig. 6C and Table 1). We conclude that the cooperation of RNase J-RhpA is not significantly affected by the K649A and K649R mutations.

### Analysis of the consequences on *H. pylori* morphology of RNase J mutations in acetylated residues

We next wanted to examine the consequences of the mutations affecting RNase J oligomerization and/or acetylation state in live *H. pylori* cells. Since RNase J is an essential protein, strains carrying Lys to Ala mutations in the 9 RNase J acetylated residues identified above were constructed with the following strategy. *H. pylori* strain B128 was transformed with plasmid pILL2157 derivatives either expressing wild type or mutant variants of RNase J under the control of an IPTG-inducible promoter. Then, the chromosomal wild type copy of RNase J was deleted by homologous recombination and replacement with a kanamycin resistance cassette.

**Fig. 5:**
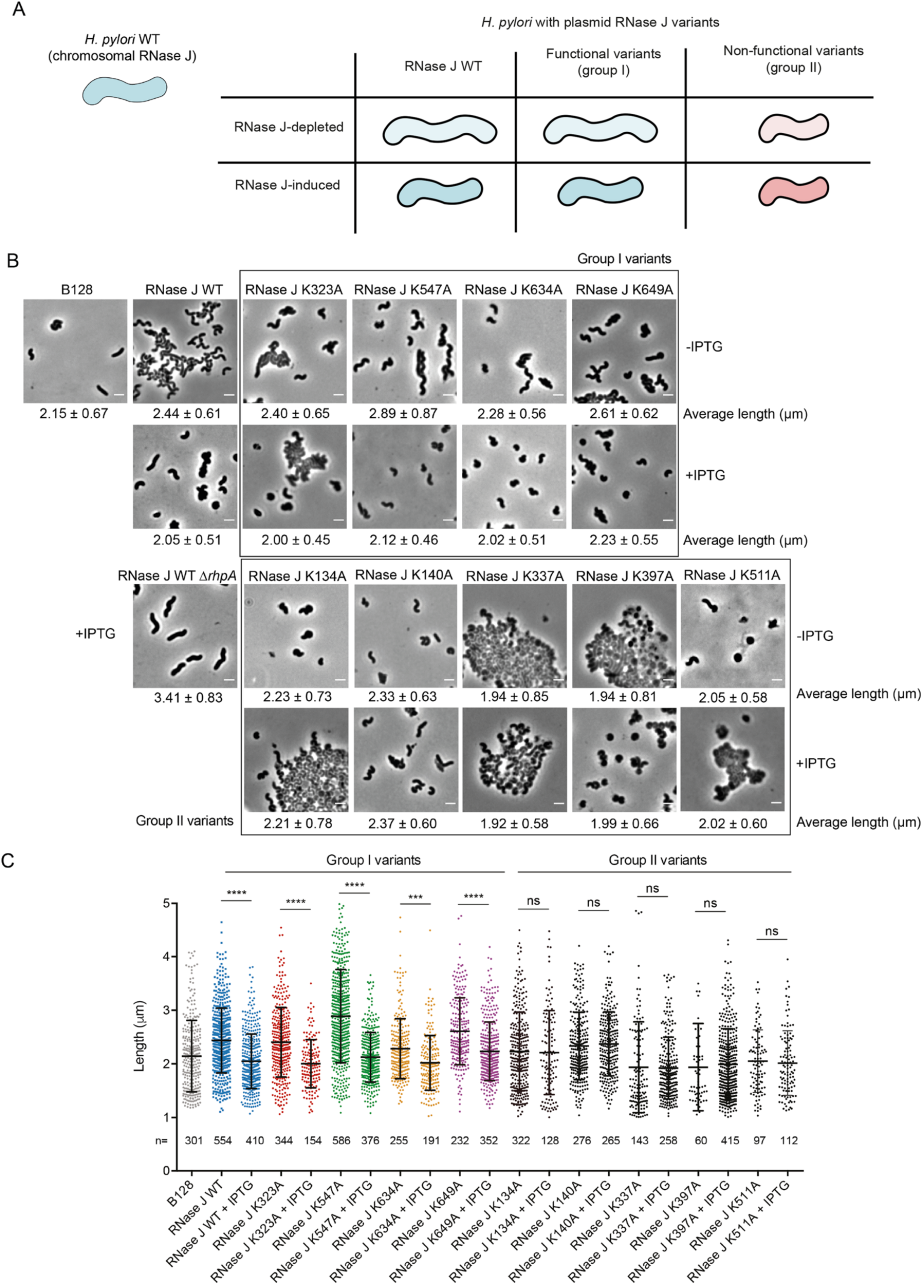
*Helicobacter pylori* cell morphology is affected by RNase J abundance and point mutations at lysine residues that are acetylated. **A** Scheme summarizing the different morphology phenotypes found in this experiment. In blue, cells displaying wild type or wild type-like functional RNase J phenotypes. In red, cells displaying non-functional RNase J phenotypes i.e., a reduced cell length that is not restored upon RNase J induction by IPTG. **B** Phase contrast microscopy images of *H. pylori ∆rnj* B128 strains expressing WT or mutant versions of RNase J from plasmid pILL2157 without (top panels) or with IPTG induction (bottom panels). The phenotype of a *H. pylori ∆rnj*-*∆rhpA* double mutant strain expressing WT RNase J from a plasmid is also shown. The scale bar represents 2 µm. Representative images of three biological replicates are shown. **C** Dot plots showing the distribution of the cell lengths of wild type and the different mutants without or with IPTG. The number of cells analyzed is indicated below each plot. Horizontal lines indicate the mean length, and the error bars are the standard deviation. ns (non-significant), ***p-value < 0.0005, ****p-value < 0.0001. The statistical test applies was one-way ANOVA with Sidak’s multiple comparisons test. Source data are provided as a Source Data file.

In *B. subtilis*, mutants deficient in RNase J1 present an altered cell morphology^25^. Therefore, the morphology of our *H. pylori* RNase J mutant strains was examined by phase contrast microscopy and quantified with the MicrobeJ ImageJ plug-in^26^. The wild type B128 strain presents cells that are relatively heterogeneous in shape and with an average length of 2.15 ± 0.67 µm. We observed that, in a strain expressing wild type RNase J from the system described above, the *H. pylori* cells were longer in the absence of IPTG [lower amount of RNase J as compared to the WT strain^8^], than upon induction with 1 mM IPTG (where the expression of RNase J is induced). The cell length was 2.44 ± 0.61 µm without IPTG and 2.05 ± 0.51 µm with IPTG (Fig. 5, see panel A for the strategy). This indicates that in *H. pylori*, RNase J is also regulating a factor required to maintain wild-type cell morphology. Interestingly, we noted that the observed phenotype is not dependent on the activity of RNase J alone but rather on the RNA degradosome complex, as IPTG induction of RNase J WT in a strain lacking RhpA fails to complement this phenotype (average length of 3.41 ± 0.83 µm) (Fig. 5B).

First, the growth curve of every mutant strain was established without and with IPTG (Supplementary Fig. 7) and Western blots were performed to verify that the amounts of the different RNase J variants proteins inside *H. pylori* were the same as the wild type RNase J protein (Supplementary Fig. 8). Then, we performed the test described above with the strains that expressed different RNase J variants and we identified two groups of mutations (Fig. 5). In the first group, the RNase J variants behaved similarly to the wild type enzyme, as their expression upon IPTG addition resulted in a reduction of bacterial length. This was the case for RNase J K323A, K547A and K649A and, to a lesser extent, RNase J K634A. In the second group, that includes variants K134A, K140A, K337A, K397A and K511A, the strains presented a strong growth defect (except K140A), and substantial cell lysis and cellular debris were observed. In these cases, the cells were short with a tendency towards a coccoid morphology that is typical of *H. pylori* cultures under stress^27^. This phenotype was particularly evident in the case of cells expressing variant K397A. For these cultures, the addition of IPTG favored bacterial growth but failed to restore the typical helical form of healthy *H. pylori* cells, suggesting that RNase J function is strongly impaired (Fig. 5 and Supplementary Fig. 7).

These results show that RNase J activity is important for the correct regulation of growth and cell morphology in *H. pylori* and that several of the residues that we found to be acetylated are important for RNase J function in vivo.

### RNase J is acetylated through a range of mechanisms in *H. pylori*

**Fig. 6:**
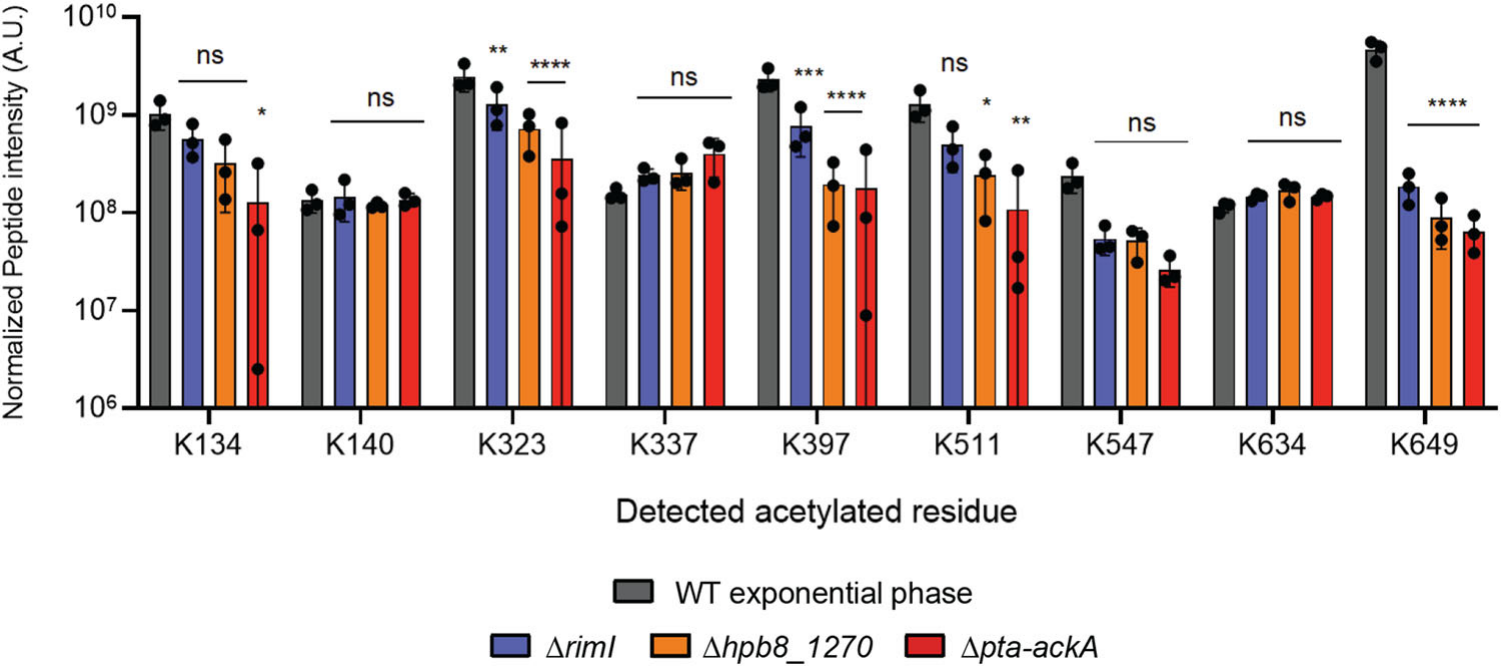
RNase J peptides containing acetylated residues extracted from different *H. pylori* strains. The normalized acetylated peptide intensities were obtained by mass spectrometry from strains expressing FLAG-tagged RNase J in a wild-type background or in a mutant strain lacking one of the different predicted acetylation mechanisms. For each condition, three individual samples were analyzed. The data are presented as bar graphs of mean values with SD, individual measurements are indicated. A.U corresponds to arbitrary units. ns (non-significant) *p*-value > 0.05, *p-value < 0.05, **p-value < 0.005, ***p-value < 0.0005, ****p-value < 0.0001. The statistical test applied was the two-way ANOVA with Dunnett’s multiple comparisons test. Source data are provided as a Source Data file.

Several pathways contribute to acetylation in bacteria^28^. We tried to elucidate which are the enzymes responsible for the different acetylation events that occur on the RNase J of *H. pylori*. Therefore, we used the *H. pylori* strain expressing an RNase J-FLAG fusion from the chromosome to introduce deletions in genes that we predicted as acetylase candidates. The RNase J-FLAG protein was pulled down from these strains and analyzed by MS as explained above. The tests were performed from three biological replicates. The acetylase candidate genes were *hpb8_615* [henceforth referred to as *rimI*, as it presents homology to *rimI* from *E. coli*^29^] and *hpb8_1270* [that contains two Gcn5-acetyltransferase (GNAT) domains]. Non-enzymatic acetylation by acetyl-CoA and acetyl-phosphate also occurs in bacteria^28,30^. The impact of this pathway was assessed in *H. pylori* by constructing a mutant of the *pta-ackA* operon (*hpb8*_667-668) that is in charge of acetyl-phosphate synthesis and metabolism. As shown in Fig. 6, we found that several pathways influence the acetylation of RNase J lysine residues. RimI is partially responsible for the acetylation of RNase J residues K323, K397 and K649. The HPB8_1270 protein is partially responsible for the acetylation of residues K323, K397, K511 and K649. Acetyl phosphate-mediated non-enzymatic acetylation participated in the acetylation of K134, K323, K397 and K511 and K649. For other residues, namely K140, K337, K547 and K634, no change in acetylation levels was detected in the mutants, suggesting that other acetylation mechanisms are likely at play in *H. pylori*. These results suggest a high redundancy of the different acetylation mechanisms that act on RNase J, particularly on residues K323, K397 and K649. Thus, multiple mechanisms participate in the acetylation of RNase J on different residues.

## Discussion

In this study, we defined the structure of the major *H. pylori* ribonuclease RNase J and its in vitro oligomerization state as well as that of its cooperative partner, the RhpA RNA helicase. We also identify in vivo acetylation patterns of RNase J and demonstrate that the corresponding residues impact on oligomerization and activity in vitro and in vivo.

Our crystal and cryo-EM structures of *H. pylori* RNase J revealed a tetrameric arrangement composed of a dimer-of-dimers. These experimental structures are in excellent agreement with predictions from the programs Robetta and Alphafold that are based on machine learning. The N-terminal region, corresponding to residues 1–136, is not resolved in the cryoEM maps. This region is not predicted to form a compact, globular structural module but rather to be intrinsically disordered^9^. Such a N-terminal segment is a distinctive feature of the RNase J homologs of the *Helicobacter* lineage, but its functional importance is not clear. Based on the behavior of other bacterial ribonucleases with intrinsically disordered domains, this domain might contribute to the capacity of RNase J to form the membrane punctate bodies or condensates observed *in H. pylori*^9^.

Like *H. pylori* RNase J, the homologs of numerous other bacterial species are also observed to form tetramers in equilibria with dimeric forms. *S. pyogenes* crystallizes as tetramers that are composed of a dimer-of-dimers^12^. Other studies showed that in vitro, RNase J can be in equilibrium between different oligomeric forms. RNase J from *D. radiodurans* is distributed between monomers and dimers^13^, whereas *Bsu*RNase J1 forms dimers and tetramers in vitro^14^ and, when mixed with *Bsu*RNase J2, heterotetramers^10^. RNase J1 from *S. aureus* forms homodimers and homotetramers in vitro^11^. In our analysis of full-length RNase J of *H. pylori* by AUC, we observe sedimentation species corresponding to both the dimer and tetramer.

Acetylation has emerged in recent years as a major post-translational regulatory mechanism in prokaryotes^16^, and a few examples of its influence in RNases and RNA-degrading enzymes have been described, including *E. coli* RNase R^18^ and RNase II^19^ and *B. subtilis* CshA^20^. Here, we found that, in *H. pylori*, as many as 9 lysine residues of RNase J were consistently acetylated, although probably not at the same time, providing evidence of protein acetylation in *H. pylori*. One of these residues is K649, which we also found to be involved in the self-interaction of RNase J by BACTH in *E. coli*. Interestingly, this residue is located at the CTD of RNase J (Supplementary Fig. 3). This region was also shown to be important for the oligomerization and exoribonucleolytic activity of the RNase J of *D. radiodurans*^13^. Therefore, we examined the role of K649 in RNase J oligomerization, by AUC, and tested the exoribonuclease activity. Three mutants were analyzed, RNase J K649A that abolishes the side chain of the lysine residue, K649R that mimics a non-acetylated state while maintaining the positive charge and K649Q that mimics an acetylated state^24^. Whereas the K649R variant maintains a sedimentation profile similar to that of the wild type enzyme, variants lacking a positive charge in K649, either K649Q or K649A, had an increased proportion of tetramer. Interestingly, we observed that the K649R variant is less active in vitro than the wild type RNase J whereas variants K649Q and K649A are more active than the K649R variant.

Our structural data indicated that residue K649 is not located at the interaction surface between the subunits of the dimer, but that it rather plays a structural role in the integrity of the overall structure (Fig. 1, Supplementary Fig. 10). We conclude that elimination of the positive charge at residue 649 leads to a higher stabilization of the tetrameric species. Electrostatic interactions of charged side chains on the surface of proteins can strongly contribute to the fold stability^31–33^. We propose that the network of surface electrostatic interactions stabilizes the CTD of RNase J, which in turn supports the homodimer. The CTD is the primary self-complementary interface of the dimer, and the interface between the core (amino acids 137–586) and between the dimer-of-dimers formed by the core both have marginal stability in isolation (Supplementary Fig. 10). Thus, changes in the charge, through acetylation, can thereby impact on stability of the CTD fold and oligomerization state.

The question naturally arises of how the catalytic activity of RNase J is affected by its oligomerization state. A clue is suggested in the overlay of the structure of *H. pylori* RNase J with the *S. coelicolor* homologue, in which there is a bound RNA substrate engaged at the active site^12^ (Fig. 1C). There are marked differences in the positions of three loops at the entrance to the catalytic site that would obstruct the RNA from following the same route in the *H. pylori* enzyme, but the nucleic acid could approach the site from the opposite rim of the entrance. The exposed peptide loop regions are positioned to accommodate the CTD. It is possible that changes in the oligomerization state, through changes in CTD stability, will impact on the presentation of those loops and access to the active site in a gating mechanism.

Altogether, our results suggest that mutations that favor the tetrameric RNase J conformation also increase the activity of RNase J of *H. pylori* in vitro. As the charge of residue 649 is important in the equilibrium between dimerization and tetramerization, acetylation of K649 might tilt the balance of the RNase J species towards the tetrameric form and potentially increase its activity. In this way, acetylation on K649 could modulate the activity of RNase J in *H. pylori* cells. Earlier studies indicate that RNase J and the RNase J-RhpA complex can act on different transcripts^2,8,34^. It can be envisaged that acetylation of K649 would therefore have a differential impact on the subset of free RNase J that is not part of a degradosome assembly.

Furthermore, we addressed the role of the acetylated residues that we identified in *H. pylori* cells. It has been described that *B. subtilis* cells lacking RNase J1 show morphology defects with a tendency to filament^25^. We thus evaluated the morphology of *H. pylori* cells expressing RNase J variants, in which each acetylated lysine was replaced by an alanine residue. We observed that cells with a reduced concentration of wild type RNase J are longer than those in which RNase J expression is induced. This revealed that RNase J, most probably indirectly, is involved in the control of *H. pylori* length. Interestingly, this phenotype does not depend on the activity of RNase J alone, but rather on the joint activity of RNase J and RhpA (or maybe in addition on RhpA alone) since the overexpression of RNase J WT in a ∆*rhpA* strain does not reduce cell length. Given that we previously showed that about 80% of *H. pylori* mRNAs are at least two times more abundant in an RNase J-depleted mutant, it is difficult to define the target mRNA that is causing the observed elongation phenotype^8^. One possibility would be the overexpression of MreC (9.3-fold upregulated in an RNase J-depleted strain), which has been shown to cause cell filamentation in *H. pylori*^35^.

Alanine replacement of the different acetylated positions of RNase J leads to different phenotypes, with five “lysine to alanine” variants out of the nine variants examined that became non-functional in this test. This strongly suggests that acetylation is indeed important for RNase J activity in *H. pylori*.

Variant K649A behaves like the wild type enzyme in this test, in agreement with the activity assays presented above and with the notion that the morphology phenotype depends on the activity of the whole RNA degradosome complex, which is not affected by whether there is a charge at this position or not. The phenotype of the K134A mutant suggests that it is affected in RNase J activity; interestingly this residue is located within the N-terminal extension of *Hp*RNase J (Supplementary Fig. 3), that we previously found to be very important for efficient RNase J activity^8^. However, it is not known how these acetylated residues impact RNase J properties in *H. pylori*. They could affect its stability, activity, oligomerization, degradosome complex formation, compartmentalization, foci formation, binding of other partners or RNA, or a combination of these. Further evidence is required to establish the physiological importance of acetylation of these amino acids in *H. pylori*.

We found that several mechanisms participate in the acetylation of the different residues of RNase J. Our results indicate that HPB8_1270 and the *H. pylori* RimI homologue indeed function as acetylases. In addition, we uncovered a role of non-enzymatic acetylation by acetyl phosphate that is produced and metabolized by the Pta and AckA enzymes. The *E. coli* RimI protein is a multifunctional acetyltransferase, that can perform ε-lysine acetylation and Nα acetylation on the L31 and S18 ribosomal proteins, respectively^29,36^, with high substrate specificity. In contrast, RimI from *Mycobacterium tuberculosis* is an Nα acetylase with more relaxed substrate specificity in vitro^37^. Here, we found that the *H. pylori* RimI targets K323, K397 and K649 of RNase J. HPB8_1270 is a protein belonging to the DUF2156 family, containing two GNAT domains. It presents homology with the BT_3689 protein from *Bacteroides thetaiotaomicron*, which contains an acetyl-CoA molecule in its active site (see PDB 2HQY), suggesting its function as an acetyltransferase. Here, we found that HPB8_1270 is involved in the acetylation of K323, K397, K511 and K649, showing that, in *H. pylori*, this protein is most likely an acetyltransferase. Lastly, acetyl phosphate-mediated acetylation, which is responsible for most acetylation events in *E. coli* and *B. subtilis*^28,30^, was important for the acetylation of K134, K323, K397, K511 and K649.

These data demonstrate that multiple mechanisms can target the same lysine residue and that there is a high redundancy in the functions of these acetylation mechanisms. Acetylation at residue K649 was strongly dependent on three such mechanisms; this redundancy could testify of a critical importance of acetylation at this position. In addition, other acetylation mechanisms could occur in *H. pylori* that might account for some of these acetylation events. More work is needed to demonstrate the mechanisms of amino acid acetylation in *H. pylori*, in particular using multiple mutants to determine how many of these mechanisms can target the different residues and to establish the influence of the different acetylation mechanisms on the activity and regulation of RNase J and the RNA degradosome in *H. pylori*.

Using Clustal Omega 1.2.4^38^, we performed an alignment of the RNase J proteins of *H. pylori* and of the closely related organism *Campylobacter jejuni*, but also of the more distant *B. subtilis*, *S. aureus*, *S. pyogenes* and *D. radiodurans*. Interestingly, most of the acetylated lysines are conserved in *H. pylori* and *C. jejuni* RNase J proteins, and some of them are also conserved in more distant organisms (Supplementary Fig. 9). The case of K511 is particularly interesting, as it is conserved in *S. pyogenes*, *B. subtilis*, *H. pylori* and *C. jejuni*, and in the case of *S. aureus* this position is a Q residue which might behave as a “permanently-acetylated” lysine. In *H. pylori*, the mutant expressing RNase J K511A variant presents a strong growth defect. The relevance of lysine acetylation in the regulation of the activity of the RNase J proteins from other organisms remains to be addressed.

In conclusion, we have established that RNase J is acetylated on multiple lysine residues through different mechanisms, and that some of its acetylated residues affect the activity of RNase J and phenotypes such as cell morphology. Furthermore, our data suggest that acetylation on K649 can influence the oligomeric state and activity of RNase J, and that the tetrameric form could be the most active one. Thus, our data suggest that acetylation is an important player in the modulation of the activity of RNase J of *H. pylori*.

## Methods

### Bacterial strains and growth conditions

The bacterial strains used in this study are summarized in Supplementary Data 2. *H. pylori* strains were derivatives of strain B128^39,40^. Plasmids (Supplementary Data 2) were constructed and amplified using *E. coli* XL1-Blue (Agilent Technologies). *H. pylori* strains were grown on Blood agar base 2 (Oxoid) plates supplemented with 10% defibrinated horse blood and with an antibiotic-antifungal cocktail composed of 2.5 µg/mL amphotericin B, 0.31 µg/mL polymyxin B, 6.25 µg/mL trimethoprim and 12.5 µg/mL vancomycin. *H. pylori* mutants were selected using 20 µg/mL kanamycin, 10 µg/mL apramycin or 6 µg/mL chloramphenicol. For liquid cultures, we used Brucella broth supplemented with 10% fetal calf serum (FCS) (Eurobio), the antibiotic-antifungal cocktail and, when necessary, chloramphenicol to select for plasmid-containing bacteria. *H. pylori* cells were grown at 37 °C under a microaerophilic atmosphere (6% O_2_, 10% CO_2_, 84% N_2_) using an Anoxomat (Mart Microbiology) atmosphere generator.

Growth curves were performed in 1 ml of Brucella broth supplemented with 10% FCS and antibiotic cocktail, in 24-well plates with a Spark microplate reader (TECAN) at 37 °C, 150 rpm and under an atmosphere containing 10% CO_2_ and measuring the optical density at 600 nm every hour.

### Plasmid preparation and transformations

A NucleoBond Xtra midi kit (Macherey-Nagel) and a QIAamp DNA minikit (Qiagen) were used for plasmid preparations for *H. pylori* transformation and genomic DNA extractions, respectively. For plasmid preparations for *E. coli* transformation and sequencing, a QIAprep Spin Miniprep kit (Qiagen) was used. PCR was carried out with either DreamTaq DNA polymerase (Thermofisher) or with Q5 High-Fidelity DNA polymerase (NEB) when the product required a high-fidelity polymerase. A list of the oligonucleotides that were used can be found in Supplementary data 3.

### Construction of *H. pylori* mutants and fusions

The cassette for the construction of the *H. pylori* RNase J-FLAG strain was constructed by the isothermal assembly technique^41^, by assembling in frame the last 500 bp of the *rnj* gene followed by the FLAG-tag, the apramycin resistance gene and the 500 bp immediately downstream from *rnj*. The resulting fragment was PCR-amplified and 1 µg of the product was introduced into *H. pylori* by natural transformation. Transformants were selected on plates with apramycin. Strains carrying the pILL2157 plasmid with the wild type *rnj* gene^2^ and its derivates with the different *rnj* mutants (constructed following standard procedures with the primers compiled in Supplementary Data 3) were transformed in the same manner with 1.5 µg of the plasmids and selected on plates with chloramphenicol. The resulting strains were then used to transform, by cassettes constructed in a similar manner, in order to delete the *rnj* gene and replace it by a kanamycin resistance gene [as in ref. ^2^]. The transformants were selected on plates containing kanamycin, chloramphenicol and 1 mM IPTG to allow the expression of the copy of *rnj* from the plasmid.

Plasmids for BACTH were constructed from plasmids pUT18 and pKNT25^42^ by inserting wild type or mutant variants of the *rnj* gene in the *Pst*I and *Kpn*I restriction sites following standard techniques using plasmids indicated in Supplementary Data 2.

Deletion of the genes of interest, construction of mutants, of fusions and/or insertion of cassettes was verified by PCR and sequencing of the region of interest.

### Purification and crystallography of RNase J

A vector co-expressing residues 21–465 of RhpA with a C-terminal 3C-HALO-his6 tag and residues 139–691 of RNase J (C1hRSEj) (from *H. pylori* strain 26695 gene HP1430, UniProt P56185) with a C-terminal hexa-histidine tag was generated by the Oxford Protein Production Facility at Harwell. *E. coli* cells were grown in terrific broth supplemented with 0.5 M NaCl and 1 mM betaine, 30 µg/mL kanamycin, 50 µg/mL carbenicillin and 20 µg/mL chloramphenicol. Cells were grown at 37 °C until optical density at 600 nm reached 0.3, then moved to 20 °C and induced with 0.2 mM IPTG (isopropyl β-D-1-thiogalactopyranoside) for 24 h. Cells were harvested by centrifugation, then suspended in 50 mM Tris pH 7.4, 50 mM HEPES (4-(2-hydroxyethyl)−1-piperazineethanesulfonic acid) pH 7.4, 500 mM NaCl, 1 mM TCEP (tris(2-carboxyethyl)phosphine), 0.05% v/v Triton X-100, 5% v/v glycerol and 10 mM imidazole and stored frozen. The suspension was thawed, passed 5 times through a cell disruptor (Avestin Emulsiflex C5 homogeniser, 1000 bars) and clarified by centrifugation at 37,000 g for 30 min, 5 °C. The supernatant was applied to a HiTrap Ni chelating column, washed with lysis buffer containing 0.01% v/v Triton X-100, and 15 mM imidazole, then eluted with a gradient in imidazole. The optimal fractions were pooled and cleaved with 3CP-GST protease cleavage, using 300 µl of 0.85 mg/mL protease to cleave C1/RSE protein isolated from 1 L culture, at 4 °C overnight. This step removes HALO-Chis-tag on RhpA. The 3CP-cleaved protein was loaded onto HALO-RESIN and the flow-through collected, which contained RNase J and RhpA. The sample was concentrated with a 30kD MWCO centrifugal concentrator and applied to a S200 SEC column equilibrated in 20 mM Tris-HCl pH 7.4, 20 mM HEPES pH 7.4, 150 mM NaCl, 5% glycerol, 0.1% v/v Triton X-100, 1 mM TCEP. The sample was concentrated with a 30 kD MWCO centrifugal concentrator to optical density at 280 nm of 3.38 and used in crystallization screens. Crystals were found in PEG I screen 0.1 M HEPES pH 7.5, 25% wt/v polyethylene glycol 1000 and only corresponded to the RNase J protein. No crystals corresponding to a RNase J-RhpA complex were identified.

Diffraction data were collected at I24 at Diamond Light source at wavelength 0.9778 Å using a Pilatus6M detector. Crystals are in space group P4_1_22 with cell dimensions a = b = 158 c = 214.26 Å. The structure was solved by molecular replacement using *Streptomyces* RNase J (PDB 3BK2) for with PHASER and the CCP4 suite^43,44^. There was no space in the lattice for the RhpA protein. The model of RNase J 139–691 was built using COOT^45^ and refined with PHENIX^46^. A homology model for the CTD, residues 587–691, was prepared using PHYRE2^47^, and the structure of the full-length protein was predicted using the ROBETTA server^21^. The CTD from the ROBETTA model was docked into the map and fitted as a rigid body and then linked to the model of the well resolved core (139–586). As the density for the CTD was poorly resolved due to disorder, the side chains were removed. A final cycle of refinement using the core only (137–586) and not the disordered CTD were used to provide the refinement parameters summarized in Supplementary Table 1A. A monomer of RNase J 139–691 occupies the asymmetric unit, and the tetramer is generated through crystallographic symmetry. Estimates of buried surface area were made using the PISA program in the CCP4 suite^48^. The model and structure factors have been deposited in the Protein Data Bank (PDB) with accession code 7PCR.

### Purification of *Helicobacter pylori* RNase J and RhpA

The DNA from *H. pylori* strain B128, encoding for RNase J was cloned into pExp-xMBP-CHis vector to result in a fusion of RNase J with N-terminal MBP tag followed by TEV protease cleavage site and C-terminal 8xHis-tag, standard PCR-based methods were used to introduce D299A mutation. The DNA encoding for RhpA was cloned into pExp-MBP vector to result in a fusion of RhpA with N-terminal 6xHis and MBP tag followed by TEV protease cleavage site and C-terminal Strep-tag. Typically, the protein was expressed in C43(DE3) strain of *E. coli* and grown in 2xYT medium supplemented with 100 µg/ml ampicillin. Protein expression was induced by addition of 0.4 mM IPTG and progressed for 16 h at 18 °C. Cells were pelleted by centrifugation, resuspended in a buffer containing 20 mM sodium phosphate pH 7.2, 500 mM NaCl, 20 mM imidazole, 0.5 mM TCEP supplemented with Complete protease inhibitor cocktail (Roche) and 250 µg/ml of DNaseI and lysed using Avestin EmulsiFlex C-5 homogenizer. Cleared lysate was loaded onto gravity flow column with 5 ml of Ni-NTA agarose (Qiagen), the resin was washed with a buffer containing 20 mM sodium phosphate pH 7.2, 500 mM NaCl, 20 mM imidazole, 0.5 mM TCEP and the protein eluted in a same buffer containing 250 mM imidazole. The eluate was loaded onto gravity flow column with 5 ml amylose resin (NEB), the resin was washed with 20 mM sodium phosphate pH 7.2, 300 mM NaCl, 0.5 mM TCEP and the protein was eluted in the same buffer with addition of 20 mM maltose. Then MBP fusion partner was cleaved off by TEV protease (produced in-house). Then the protein of interest was further purified by heparin-affinity chromatography by loading onto HiTrap Heparin 5 ml column (Cytiva) and eluting in a linear gradient of 20 mM HEPES-NaOH pH 7.2 and 100–1000 mM NaCl. Finally, the protein was purified by size-exclusion chromatography using Superose 6 Increase 10/300 column (Cytiva) equilibrated in gel filtration buffer containing 20 mM sodium phosphate pH 7.2, 300 mM NaCl, 0.5 mM TCEP.

### Mass photometry

Mass photometry experiments were performed using Refeyn TwoMP mass photometer (Refeyn). Borosilicate microscope coverslips (24 ×50 mm 1.5 H, Marienfeld) were cleaned using ethanol and MilliQ water (3x) and dried under nitrogen stream. Silicone gaskets (6 wells, 3 mm diameter x 1 mm depth, Refeyn) were placed onto clean coverslips. Before data acquisition, coverslip wells were loaded with 18 μL of buffer (20 mM sodium phosphate pH 7.2, 300 mM NaCl), followed by focusing the instrument on the glass-liquid interface. Before measurements the protein concentration was adjusted to 500 nM. Typically, mass photometry experiment was initiated by jump-diluting 2 μL of a protein solution directly in the buffer-loaded well to reach a final concentration of 50 nM, followed by recording for 60 s. The data were processed in DiscoverMP program (Refeyn) by combining measurements of 3000 frames into a single mass histogram and applying previously established calibration data obtained using standard samples with known molecular weight measurements [BSA (66 kDa), IgG (150 kDa) and thyroglobulin (660 kDa)].

### CryoEM of RNase J

The protein mixture of RNase J (1 µM) and RhpA (2 µM) in a buffer containing 20 mM sodium phosphate pH 7.2, 500 mM NaCl, 0.5 mM TCEP was used for grids preparation. Three μl of the sample was applied to Quantifoil R1.2/1.3 grids that had been pretreated by glow-discharge (0.29 mbar, 25 mA, 1 min, Pelco Easiglow glow discharger), and excess sample was blotted away and frozen in liquid ethane, blot force −4 to 0, blot time 3 s, Vitrobot Mark IV (Thermo Fischer). The grids were screened on a 200 kV Talos Arctica (FEI) (Cryo-EM facility, Department of Biochemistry, University of Cambridge) and the movies were recorded on the same 200 kV Talos Arctica machine with a Falcon III (Thermo Fischer, Cryo-EM facility, Department of Biochemistry, University of Cambridge). Datasets were pre-processed with Warp^49^. Particle sets were optimized in CryoSparc^50^ via repetitive 2D classifications and heterogeneous refinements. Only 34061 particles were found useful for ab-initio modeling. Further extensive classifications in 2D were used to classify particles with non-uniform refinement in cryoSPARC^51^ and global- and per particle CTF refinements. Although a mixture of the full-length RNase J with RhpA (1:2) was used as a sample, we were unable to identify a density that would correspond to RhpA. On the grids, RNase J protein embedded into vitreous ice with a preferred orientation, limiting the final resolution to 4.1 Å (Supplementary Fig. 1). The RNase J Cryo-EM data collection, refinement and validation statistics are presented in the Supplementary Table 1B. The RNase J cryoEM image processing procedures is summarized in Supplementary Fig. 1. The atomic coordinates and cryo-EM density map of RNase J have been deposited in the RCSB PDB with the accession code 8CGL and in the Electron Microscopy Data Bank (EMDB) with the accession code EMD-16647.

### Bacterial two-hybrid (BACTH)

BACTH assays were carried out in *E. coli* strain BTH101^42^. Briefly, strains carrying derivatives of the two vectors, pUT18 and pKNT25, were grown overnight in 1 mL LB with 40 µg/mL kanamycin, 100 µg/mL ampicillin and 0.1 mM IPTG. The OD at 600 nm of the resulting cultures was measured in a TECAN plate reader and the beta-galactosidase activity was calculated by mixing 500 µL of the cultures with 500 µL of buffer Z, 100 µL of chloramphenicol and 50 µL of 0.1% SDS, vortexing the cells and then adding 200 µL of 4 mg/mL ONPG. The reactions were incubated at 28 °C until they turned yellow and the reactions were stopped by adding 500 µL of 1 M Na_2_CO_3_. Samples were centrifuged for 5 min at 30,678 x *g* and the OD at 420 and 550 nm of the upper fraction was measured in a TECAN plate reader. The beta-galactosidase activity (Miller units) was calculated as previously described^52^.

### Protein expression and purification for RNase activity tests

Wild type and variant versions of RNase J and RhpA were cloned into a pET28a+ vector in *E. coli* XL1-Blue and then transformed in *E. coli* Bli5 for expression. The strains were grown in 800 mL cultures in LB with 40 µg/mL kanamycin and 25 µg/mL chloramphenicol and, once they reached an OD of 0.5–0.8, were induced with 1 mM IPTG. The cultures were incubated at 30 °C and 160 rpm during 3 h. The cells were collected by centrifugation at 2504 x *g* in an Avanti J–E centrifuge (Beckman Coulter) with a JA-14 rotor at 4 °C during 30 min and pellets were frozen at −80 °C until further processing.

Pellets were thawed on ice and resuspended in lysis buffer containing 25 mM Tris-HCl pH 7.8 for RNase J and pH 8 for RhpA, 500 mM NaCl, 10% glycerol, 20 mM imidazole, 1 mM dithiothreitol (DTT), 50 U benzonase and protease inhibitor cocktail (c0mplete EDTA-free, Roche). Cells were lysed by sonication and centrifuged for 30 min at 3041 x *g* in a 5810 R centrifuge (Eppendorf) at 4 °C to eliminate cellular debris. The supernatant was then incubated overnight at 4 °C and under agitation with 2 mL Ni-NTA resin (50% v/v, Thermofisher) pre-equilibrated with lysis buffer.

Resins were packed onto Poly-Prep Chromatography columns (Bio-Rad) and washed with 10 volumes of 25 mM Tris-HCl at the appropriate pH, 300 mM NaCl, 1 mM DTT and 20 mM imidazole, 10 volumes of the same buffer with 50 mM imidazole and 10 volumes of the same buffer with 100 mM imidazole. The elution was carried out with an elution buffer containing 25 mM Tris-HCl at the appropriate pH, 300 mM NaCl, 1 mM DTT and 500 mM imidazole, in 1 mL fractions. Aliquots of the resulting fractions were run on a 4–20% Mini-Protean TGX stain-free precast protein gel (Bio-Rad) at 200 V for 30 min and stained by Imperial protein stain (ThermoFisher). After destaining, the corresponding purest fractions were chosen, mixed and concentrated using Vivaspin 20 tubes (MWCO 30,000 Da, GE Healthcare) down to 1 mL volumes. The buffer was replaced using G-25 columns (PD MidiTrap G-25, GE Healthcare) with 25 mM Tris-HCl, 300 mM NaCl, 1 mM DTT and 10% glycerol. Samples were aliquoted and frozen at −80 °C.

### Analytical ultracentrifugation

Samples of His-tagged purified proteins were thawed on ice and the buffer was replaced using G-25 columns (PD MiniTrap G-25, GE Healthcare) with 25 mM Tris-HCl pH 8, 300 mM NaCl and 0.5 mM Tris(2-carboxyethyl)phosphine (TCEP, Sigma). Appropriate amounts of the different proteins were mixed and diluted in the same buffer with a final volume of 300 µL that was charged into 2 sector AUC cells. The centrifugation was performed for 16 h on an Optima analytical XL-I ultracentrifuge (Beckman-Coulter) with an 8-hole An-50 Ti rotor, at 157,772 x *g* and 20 °C, measuring the optical density at 230 nm in order to determine the sedimentation profile of the protein. The data was analyzed with the Sedfit v16.1c software (NIH) using a diffusion deconvoluted continuous sedimentation coefficient distribution c(s) with one discrete component model^53^. The model was chosen in order to take in consideration the signal due to TCEP absorption. The presented graphs correspond to a resolution of 300 points with a confidence interval of 0.9. For the individual proteins, the sedimentation coefficient independent of the concentration (S_0_) was calculated.

The partial specific volume ($\bar{\nu }$) of the proteins was estimated by the software Sednterp^54^ based on the amino acid sequence. This software was also used for the calculation of the density and viscosity of the buffer used throughout the experiments. Calculation of sedimentation coefficients for protein structures was performed with the program Hydropro^55^. For comparison, the profile of each analysis was normalized to 1 for the highest peak.

### Circular dichroism

The near-UV absorption spectra (195–260 nm) were measured, in the same buffer as for the AUC experiments, by circular dichroism with a Circular Dichroism Spectrometer Model 215 (Aviv instruments) using 0.2 mm optical path cells with 5 mM of RNase J WT and K649A mutant. The proportions of the different secondary structure elements were determined with the BeStSel software^56,57^ based on the amino acid sequence.

### Ribonuclease activity tests

The endoribonuclease and exoribonuclease activities were measured using 6-carboxyfluorescein (6FAM)-labeled RNA substrates either in 5’ or in 3’ as listed in Supplementary Data 3. 500 nM RNA substrate were incubated with 500 nM His-tagged protein in a total volume of 10 µL with a buffer containing 50 mM Tris-HCl pH 7.8, 100 mM NaCl, 8 mM MgCl_2_, 0.1 mM and 0.1 mg/mL bovine serum albumin (BSA). The reactions were carried out at 37 °C for 0–30 min for the exoribonuclease activity tests and 0–75 min for endoribonuclease activity tests. Reactions were stopped by adding 98% formamide and 10 mM ethylene diamine tetraacetic acid (EDTA) and by incubating for 15 min at 95 °C.

The degradation products were run on a Mini-Protean TBE-Urea gel (Bio-Rad) that was pre-run for 60 min at 200 V. The samples were run for 5 min at 50 V and then for 30 min at 200 V. Gels were visualized on a Chemidoc MP Imaging System (Bio-Rad) with 0.2 s exposure at 488 nm. The gels were then stained with ethidium bromide to reveal the molecular weight marker (ssRNA molecular weight marker, Takara). Experiments were performed in triplicate and images were quantified ImageLab (Bio-Rad, version 6.1). Decay coefficients, λ, were calculated with the formula $N\left(t\right)={N}_{0}{e}^{-\lambda t}$, where *N(t)* is the amount of substrate at time *t* and *N*_*0*_ is the initial amount. The half-lives, *t*_*1/2*_ were then calculated with the formula

$${t}_{1/2}=\frac{{Ln}(2)}{\lambda }$$

### Phase contrast microscopy

Phase contrast microscopy was performed with an Axio Observer microscope (Zeiss) equipped with an Axiocam camera with X100 magnification. Image acquisition was performed with the axiovision SE64 software. Images were cropped and adjusted using FiJi-ImageJ v2.0.0 software. The length of wild type cells and that of cells expressing wild type or mutant versions of RNase J from a plasmid, with or without 1 mM IPTG, was measured by using the MicrobeJ plugin of ImageJ^26^ with the following parameters: cell area above 0.7 µm^2^, minimal length of 1 µm and cell width between 0.4 and 1 µm.

### Pulldown and mass spectrometry

*H. pylori* cultures of strains expressing FLAG-tagged RNase J were grown in 15 mL of Brucella medium until an OD_600 nm_ of 0.6–1.2 for exponential phase cultures. Cells were pelleted by centrifugation for 10 min at 4 °C at 2504 x *g* and resuspended in PBS to an OD_600 nm_ of 10. Cells were lysed by sonication and cell debris were eliminated by centrifugation for 10 min at 26,452 x *g* and 4 °C. The supernatant constitutes the total extract fraction.

Pulldowns were performed using 2 µL of monoclonal anti-FLAG M2 antibodies produced in mice (F3165, Sigma) per reaction and 50 µL of magnetic protein G-coated Dynabeads according to the supplier’s instructions (Dynabeads Protein G for Immunoprecipitation, 10003D, ThermoFisher). Proteins were eluted in the denaturing mode and run in a 4–20% Mini-Protean TGX stain-free precast protein gel (Bio-Rad) at 200 V for 30 min, stained by Imperial protein stain (ThermoFisher) and destained.

Each band corresponding to RNase J-FLAG (number of biological replicates: 6 for the WT, 3 for ∆*rimL*, 3 for ∆*hpb8_1270*, and 3 for ∆*pta-ackA*) was cut and washed several times in 50 mM ammonium bicarbonate (ABC)-acetonitrile (ACN) (1:1) for 15 min at 37 °C. Disulfide bonds were reduced with 10 mM DTT and cysteines alkylated with 55 mM chloroacetamide. Trypsin (V5111, Promega) digestion was performed overnight at 37 °C in 50 mM ABC. Peptides were extracted from the gel by two incubations in 50 mM ABC-ACN-formic acid (FA) (50:50:0.5) for 15 min at 37 °C. After ACN evaporation in a Speed-Vac, resulting peptides were desalted with stage-tip^58^ using C18 Empore disc and eluted with 80% ACN, 0.1% FA. Peptides were resuspended in 2% ACN, 0.1% FA prior to liquid chromatography coupled to mass spectrometry (LC-MS/MS) analysis.

For LC-MS/MS, a nanochromatographic system (Proxeon EASY-nLC 1200 - ThermoFisher Scientific) was coupled on-line to a Q Exactive^TM^ Plus Mass Spectrometer (ThermoFisher Scientific) using an integrated column oven (PRSO-V1 - Sonation GmbH, Biberach, Germany). For each sample, peptides were loaded on an in-house packed 26 cm nano-HPLC column (75 μm inner diameter) with C18 resin (1.9 μm particles, 100 Å pore size, Reprosil-Pur Basic C18-HD resin, Dr. Maisch GmbH, Ammerbuch-Entringen, Germany) after an equilibration step in 100 % solvent A (H_2_O, 0.1% FA).

Peptides were eluted with a multi-step gradient using 2–7 % solvent B (80% ACN, 0.1% FA) during 5 min, 7–23% during 70 min, 23–45% during 30 min and 45–95% during 5 min at a flow rate of 300 nL/min over 132 min. Column temperature was set to 60 °C.

MS data were acquired using Xcalibur software using a data-dependent Top 10 method with a survey scans (300–1700 m/z) at a resolution of 70,000 and a MS/MS scans (fixed first mass 100 m/z) at a resolution of 17,500. The AGC target and maximum injection time for the survey scans and the MS/MS scans were set to 3E6, 20 ms and 1E6, 60 ms respectively. The isolation window was set to 1.6 m/z and normalized collision energy fixed to 28 for HCD fragmentation. We used a minimum AGC target of 1E4 for an intensity threshold of 1.7E5. Unassigned precursor ion charge states as well as 1, 7, 8 and >8 charged states were rejected and peptide match was disable. Exclude isotopes was enabled and selected ions were dynamically excluded for 30 s.

Raw data were analyzed using MaxQuant software version 1.6.6.0^59^ using the Andromeda search engine^60^. The MS/MS spectra were searched against a UniProt *Helicobacter pylori* [strain B8^39,40^] database (download in 24/06/2019, 1719 entries) and tagged RNase J. Usual known MS contaminants and reversed sequences of all entries were included.

Andromeda searches were performed choosing trypsin as specific enzyme with a maximum number of five missed cleavages. Possible modifications included carbamidomethylation (Cys, fixed), oxidation (Met, variable), Nter acetylation (variable) and K acetylation (Lys, variable) for in-gel peptides. The mass tolerance in MS was set to 20 ppm for the first search then 4.5 ppm for the main search and 20 ppm for the MS/MS. Maximum peptide charge was set to seven and five amino acids were required as minimum peptide length.

A false discovery rate cutoff of 1% was applied at the peptide and protein levels.

Raw files and MaxQuant files (msms.txt) were processed in Skyline-daily 20.1.9.268^61^ to generate MS1-XIC and perform peak integration for each K-acetylated peptide. Normalization across samples was performed by using the mean of the ratios from non-modified peptides (Supplementary data 1).

The MS data have been deposited to the ProteomeXchange Consortium via the PRIDE partner repository^62^ with the dataset identifier PXD027670.

### Statistical analyses

Statistical analysis was performed using Prism 9. We applied one-way ANOVA with Dunnett’s multiple comparisons test in Fig. 3A, one-way ANOVA with Sidak’s multiple comparisons test in Fig. 5C and two-way ANOVA with Dunnett’s multiple comparisons test in Fig. 6.

### Reporting summary

Further information on research design is available in the Nature Portfolio Reporting Summary linked to this article.

### Supplementary information

Supplementary Information

Description of Additional Supplementary Files

Supplementary Data 1

Supplementary Data 2

Supplementary Data 3

Reporting Summary

### Source data

Source Data

## Data Availability

The X-ray model and structure factors of RNase J data generated in this study have been deposited in the RCSB Protein Data Bank (PDB) database with accession code 7PCR. The atomic coordinates and cryo-EM density map of RNase J have been deposited in the PDB with the accession code 8CGL and in the Electron Microscopy Data Bank (EMDB) with the accession code EMD-16647. The mass spectrometry data generated in this study are available at the PRIDE repository with the dataset identifier PXD027670. Source data are provided with this paper.
