# Supplementary material for: Acetylation regulates the oligomerization state and activity of RNase J, the Helicobacter pylori major ribonuclease
Source: Nat Commun. 2023 Dec 6;14:8072. doi: 10.1038/s41467-023-43825-8 (PMC10700544; doi:10.1038/s41467-023-43825-8)
Supplement: Supplementary file 1 — Supplementary Information [file 41467_2023_43825_MOESM1_ESM.pdf]

Supplementary Information

Supplementary Figure 1. RNase J cryoEM image processing procedures

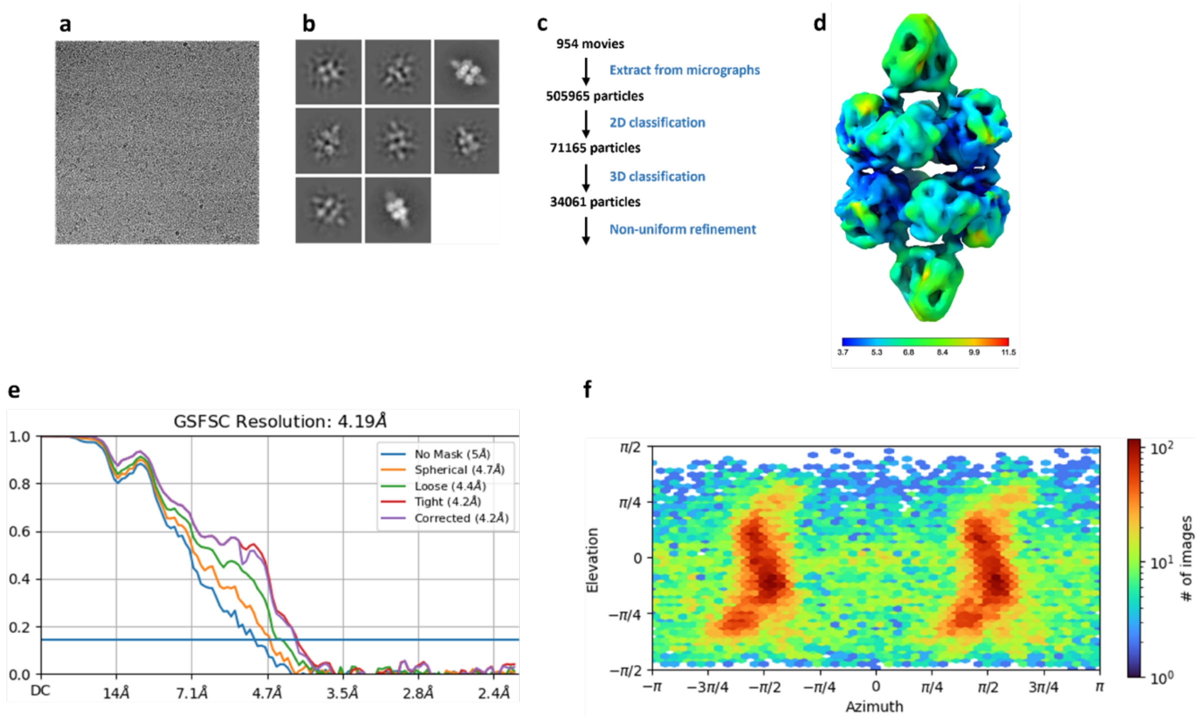

a, Representative micrograph. **b**, Representative 2D class averages obtained from reference-free 2D classification. **c**, Classification and refinement procedures used in the study. **d**, The map colored to local resolution. **e**, A global resolution estimate with Fourier shell correlation (FSC) cut-off. **f**, A representation of the angular distribution of particles used in the final reconstruction.

Supplementary Figure 2. Mass photometry analysis of purified RNase J and RhpA proteins.

A

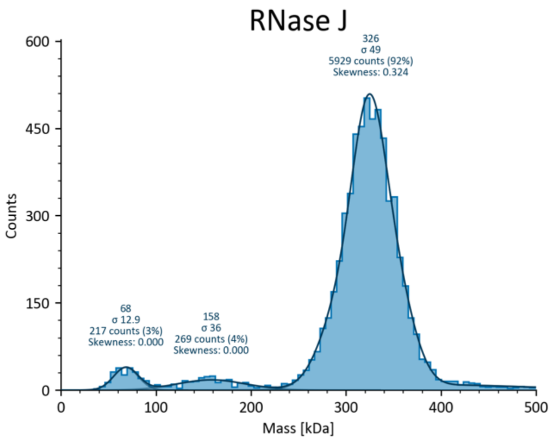

B

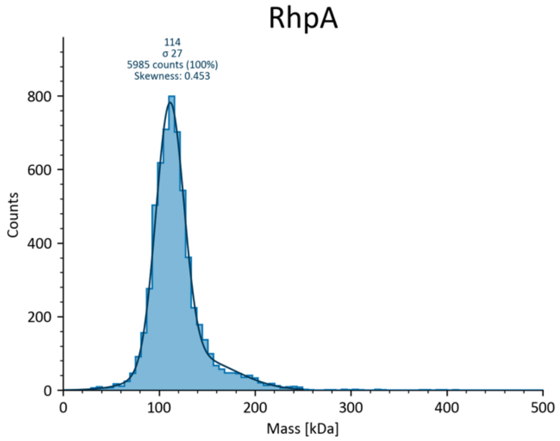

C

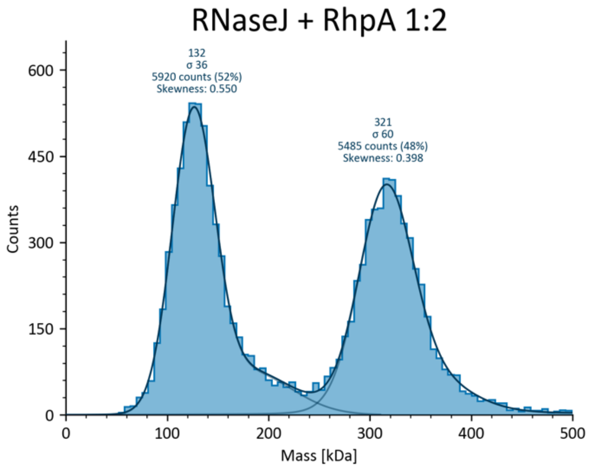

(A) The mass distribution profile for purified *H. pylori* RNase J. The masses in kilodaltons are provided above each peak, with the error estimate, the event counts for the signal and the distribution skew. The calculated masses for RNase J are 79.1 kDa, 158.2 kDa, and 316.4 for the monomer, dimer and tetramer, respectively. **(B)** The mass distribution profile for *H. pylori* helicase RhpA. The calculated Mw for RhpA is 57.3 kDa, and for the dimer 114.6 kDa. **(C)** The mass profile for the 1:2 mixture of RNase J and RhpA. The profile appears to be the sum of those for the individual

components, suggesting that the two proteins do not interact strongly under the tested solution conditions. Source data are provided as a Source Data file.

Supplementary Figure 3. Linear map of RNase J showing its domains and the positions of the different acetylated lysine residues detected in this study. NTD: N-terminal domain, CTD: C-terminal domain.

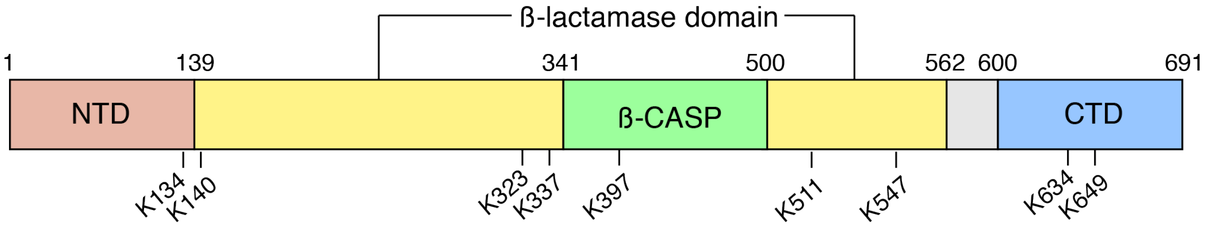

Supplementary Figure 4. Coomassie blue-stained gels presenting the purification of His-tagged wild-type RNase J and RhpA proteins and of the different RNase J variants used in this study. M: molecular weight marker; NI: non-induced lysate; I: induced lysate; FT: flowthrough. Source data are provided as a Source Data file.

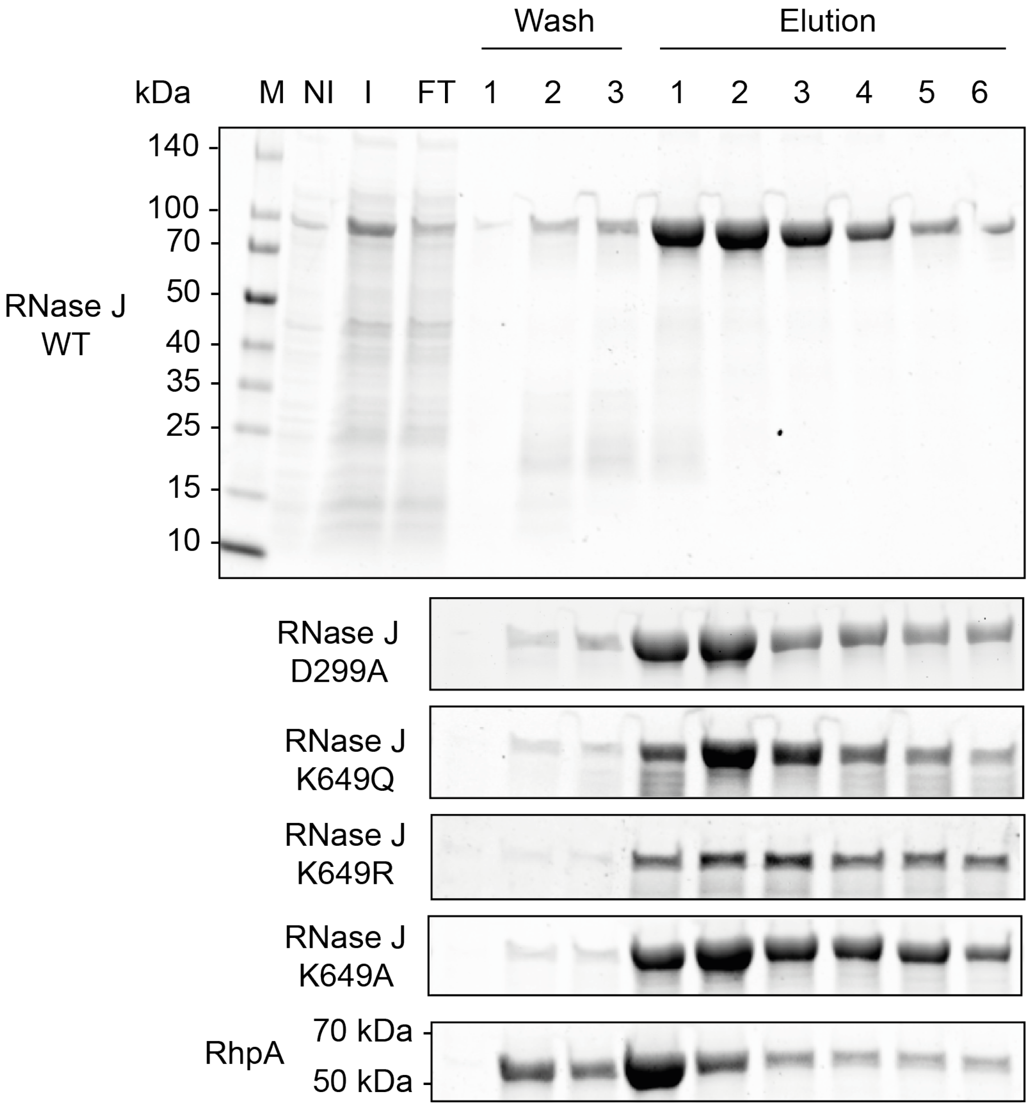

Supplementary Figure 5. Circular dichroism spectra of RNase J WT and variant K649A. Source data are provided as a Source Data file.

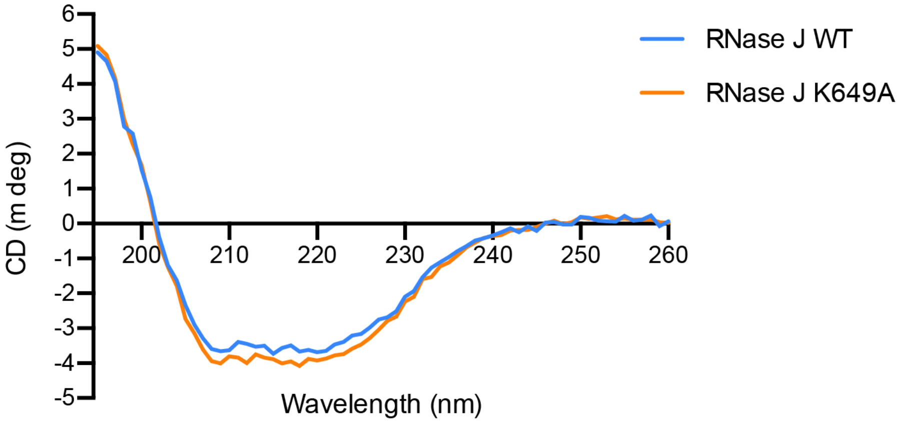

Supplementary Figure 6. (A) Secondary structure of the 45-nt RNA substrate labeled with 6-carboxyfluorescein (6-FAM) at its 3'-end used to assess the activity of the RNase J-RhpA complex. (B) Activity of the RNase J using RNase J WT and the different mutants (D299A, K649A, K649R and K649Q) over the course of 75 min. (C) Activity of the RNase J-RhpA functional complex using RNase J WT and the different mutants (D299A, K649A, K649R and K649Q) over the course of 30 min. Experiments were performed in independent triplicates. Source data are provided as a Source Data file.

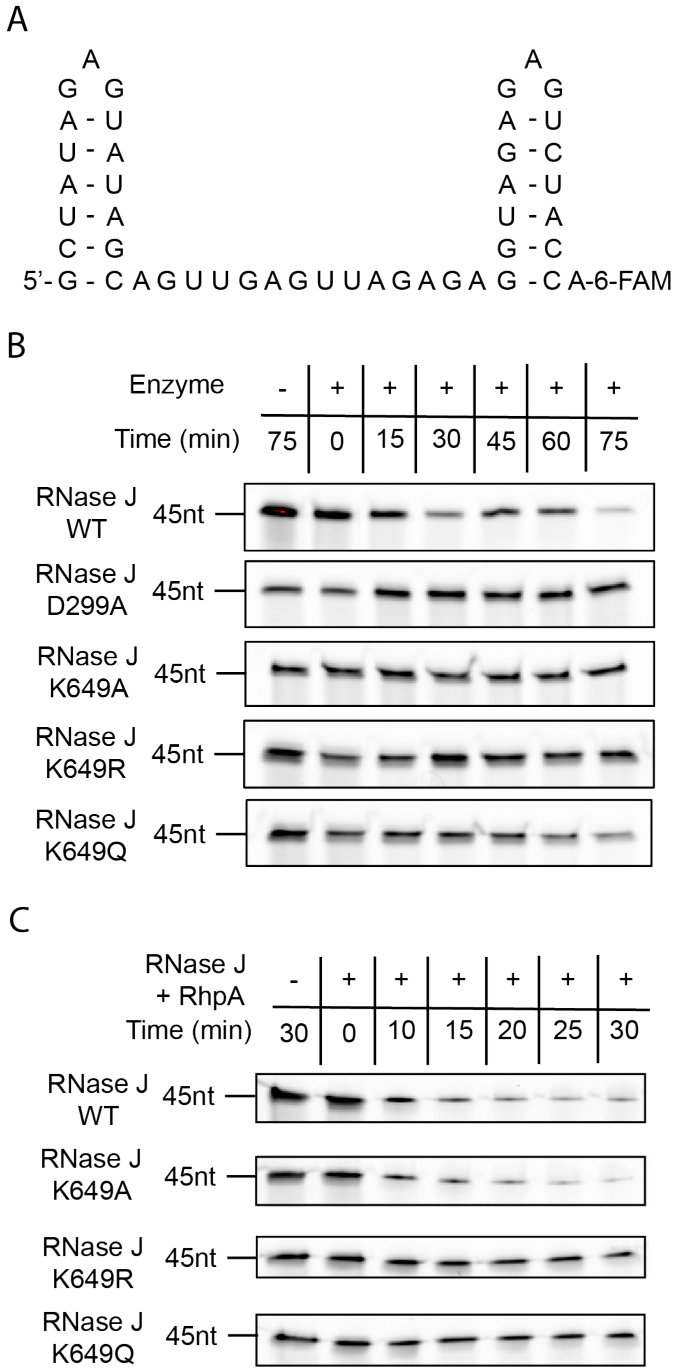

Supplementary Figure 7: Growth curves of *H. pylori* strains expressing RNase J WT or alanine-replacement mutants of the different acetylated lysines, from the pILL2157 plasmid under the control of an IPTG-inducible promoter. Growth curves are classified according to the group to which the mutants belong depending on their phenotypes, in group I and group II variants, wild type condition is shown in blue in each growth curve (see Fig. 5).

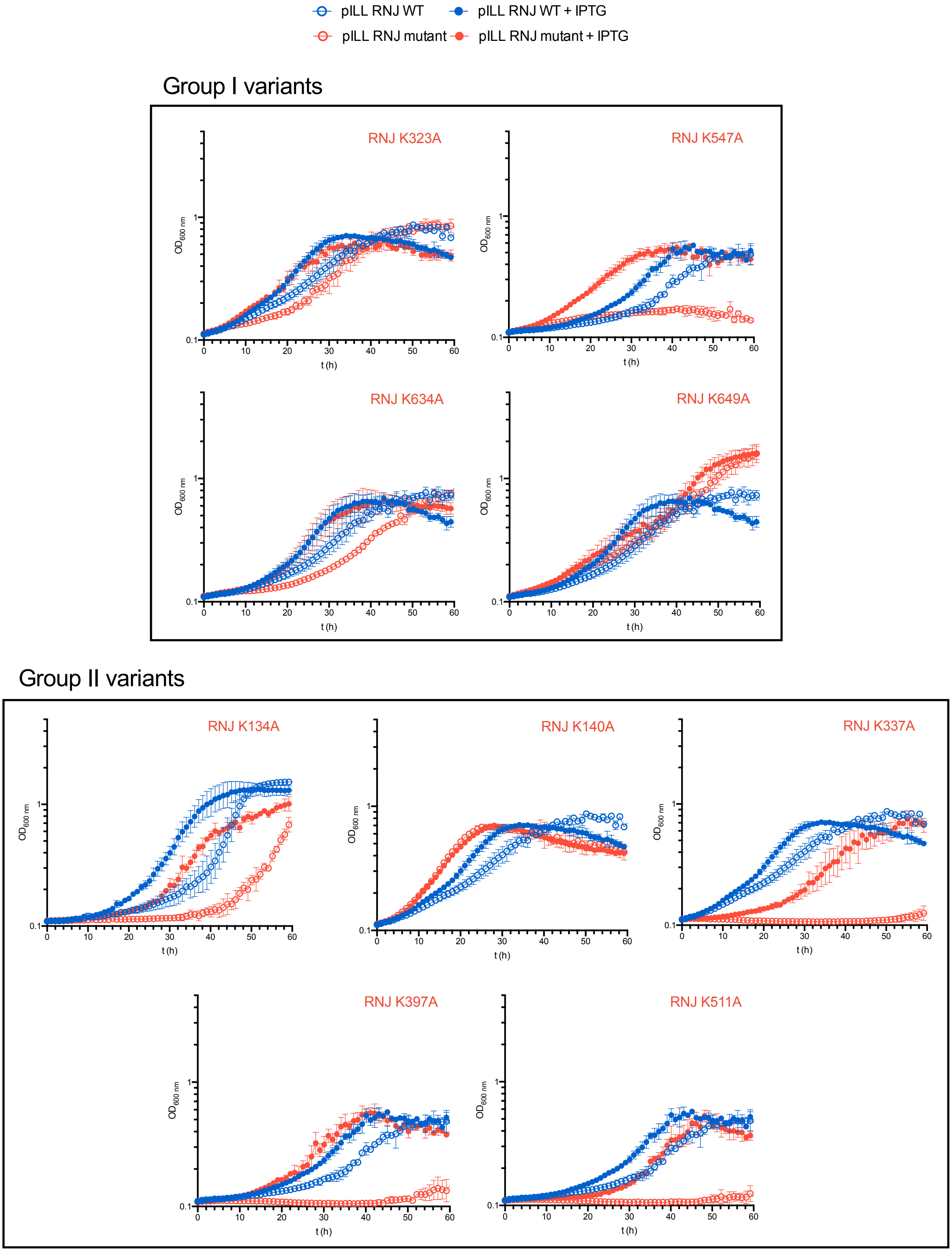

Supplementary Figure 8: Anti-RNase J Western blot showing that the levels of the different variants of RNase J are not significantly different from the wild type (WT) protein upon induction in exponentially growing *H. pylori*. B128 is the parental strain. Western blots were performed as we previously published (Tejada-Arranz *et al. MBio* 2020) with same amount of protein in each lane. Source data are provided as a Source Data file.

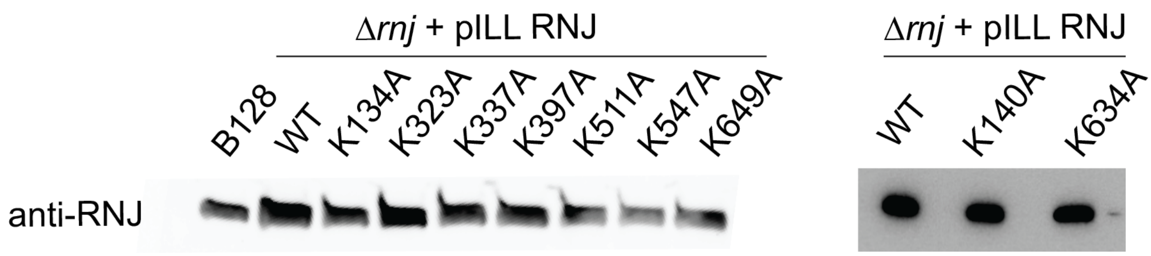

Supplementary Figure 9: Alignment of the RNase J proteins from *H. pylori*, *C. jejuni*, *S. pyogenes*, *D. radiodurans* and the RNases J1 from *B. subtilis* and *S. aureus*. Highlighted in yellow are the acetylated lysine residues from *H. pylori* RNase J and their possible counterparts in the other RNase J sequences.

```
CLUSTAL O(1.2.4) multiple sequence alignment


DraRNase      ------------------------------------------------------------  0
SpyRNase      ------------------------------------------------------------  0
BsuRNase      ------------------------------------------------------------  0
SauRNase      ------------------------------------------------------------  0
HpRNase       MTDNNHYENNESNENSSENSKVDEARAGAFERFTNRKKRFRENAQKNGESSHHEAPSHHK  60
CpyRNase      ---------------------MNEENKTPVAERTNRKHRYKHH-----------------  22


DraRNase      ------------------------------------------------------------  0
SpyRNase      ------------------------------------------------------------  0
BsuRNase      ------------------------------------------------------------  0
SauRNase      ------------------------------------------------------------  0
HpRNase       KEHRPNKKPNNHHKQKHAKTRNYAKEELDSN----KV------EGVTEILHVNERGTLGF  110
CpyRNase      ---RD-----NLKKQANANTQNVQANEVATDIAESEVKKPKKRKHKNGNNTVKISGNEGW  74


DraRNase      --------------MTRPEQPRPESADLPAPTLEVIPLGGMGEIGKNITVFRYGDEIVVV  46
SpyRNase      ----------------------MTNISLKPNEVGVFAIGGLGEIGKNTYGIEYQDEIIIV  38
BsuRNase      ------------------------MKFVKNDQTAVFALGGLGEIGKNTYAVQFQDEIVLI  36
SauRNase      ------------------------MKQLHPNEVGVYALGGLGEIGKNTYAVEYKDEIVII  36
HpRNase       HKELKKGVETNNKIQVEHLNPHYKMNLNSKASVKITPLGGLGEIGGNMMVIETPKSAIVI  170
CpyRNase      QKDMQASIEANRASHELRLNPLKYL-NSSEHKIKITPLGGLGEIGGNMTVFETDNDAIIV  133
                                                :  :**:**** *   ..  .. :::

DraRNase      DGGLAFPKAHQMGIDLIVPRIDYLLEHQDKIKGWILTHGHEDHIGGLPYIFARLPRVPVY  106
SpyRNase      DAGIKFPEDDLLGIDYVIPDYSYIVDNLDRVKALVITHGHEDHIGGIPFLLKQA-NIPIY  97
BsuRNase      DAGIKFPEDELLGIDYVIPDYTYLVKNEDKIKGLFITHGHEDHIGGIPYLLRQV-NIPVY  95
SauRNase      DAGIKFPDDNLLGIDYVIPDYTYLVQNQDKIVGLFITHGHEDHIGGVPFLLKQL-NIPIY  95
HpRNase       DAGMSFPKEGLFGVDILIPDFSYLHQIKDKIAGIIITHAHEDHIGATPYLFKEL-QFPLY  229
CpyRNase      DIGMSFPSESMHGVDILIPDFDYIRKIKQKVRGIIITHAHEDHIGAVPYFFKEF-QFPIY  192
              * *: **.    *:* ::*   *: .  ::: . ..:**.******. *::: .  ..*:*

DraRNase      GLPLTLALVREKLSEFGLQDVDLR-EVTYGDEVRFGQSFVAEFFCMTHSIPDNAGYILKT  165
SpyRNase      AGPLALALIRGKLEEHGLWREATVYEI-NHNTELTFKNMSVTFFKTTHSIPEPVGIVIHT  156
BsuRNase      GGKLAIGLLRNKLEEHGLLRQTKLNII-GEDDIVKFRKTAVSFFRTTHSIPDSYGIVVKT  154
SauRNase      GGPLALGLIRNKLEEHHLLRTAKLNEI-NEDSVIKSKHFTISFYLTTHSIPETYGVIVDT  154
HpRNase       GTPLSLGLIGSKFDEHGLKKYRSYFKIVEKRCPISVGEFIIEWIHITHSIIDSSALAIQT  289
CpyRNase      ATPLPLGMISNKFEEHGLKAHRSYFRPIEKRKLYEIGDFEIELIHITHSIIDASALVIIT  252
              .  * :.::  *:.*. *                     **** :  .  : *

DraRNase      PVGDVLHTGDFKIDPDVGTGAGIVSDLERVEQAGKDGVLLLISDSTNAERPGHTPSEAEI  225
SpyRNase      PQGKIICTGDFKFDFTPV---GDPADLQRMAALGEEGVLCLLSDSTNAEIPTFTNSEKVV  213
BsuRNase      PPGNIVHTGDFKFDFTPV---GEPANLTKMAEIGKEGVLCLLSDSTNSENPEFTMSERRV  211
SauRNase      PEGKVVHTGDFKFDFTPV---GKPANIAKMAQLGEEGVLCLLSDSTNSLVPDFTLSEREV  211
HpRNase       KAGTIIHTGDFKIDHTPV--DNLPTDLYRLAHYGEKGVMLLLSDSTNSHKSGTTPSESTI  347
CpyRNase      KAGTILHTGDFKIDHTPI--DGYPTDLNRLAYYGERGVLCMLSDSTNSYKEGITKSESSV  310
                * :: *****:*       .  ::: ::   *: **: ::*****:     * **  :

DraRNase      ARNLEEIIKGCRGRVFLTTFASQVYRIQNILDLAHRQGRRVVMEGRSMIKYAQAAQATGH  285
SpyRNase      GQSILKIIEGIHGRIIFASFASNIYRLQQAAEAAVKTGRKIAVFGRSMEKAIVNGIELGY  273
BsuRNase      GESIHDIFRKVDGRIIFATFASNIHRLQQVIEAAVQNGRKVAVFGRSMESAIEIGQTLGY  271
SauRNase      GQNVDKIFRNCKGRIIFATFASNIYRVQQAVEAAIKNNRKIVTFGRSMENNIKIGMELGY  271
HpRNase       APAFDTLFKEAQGRVIMSTFSSNIHRVYQAIQYGIKYNRKIAVIGRSMEKNLDIARELGY  407
CpyRNase      GKTFDAIFATSKGRVIMSTFSSNIHRVYQAIERGVKHGRKVCVIGRSMERNLWTAIELGY  370
              .  .  ::    **:::::*:*:::*: :  : . : .*::   ****       .   *:

DraRNase      MN-PPEPFLTSEEVGELQDQQVLFVCTGSQGQPMAVLGRLAFGTHAKIALRRGDTVILSS  344
SpyRNase      IKVPKGTFIEPSELKNLHASEVLIMCTGSQGESMAALARIANGTHRQVTLQPGDTVIFSS  333
BsuRNase      INCPKNTFIEHNEINRMPANKVTILCTGSQGEPMAALSRIANGTHRQISINPGDTVVFSS  331
SauRNase      IKAPPETFIEPNKINTVPKHELLILCTGSQGEPMAALSRIANGTHKQIKIIPEDTVVFSS  331
HpRNase       IHLPYQSFIEANEVAKYPDNEVLIVTTGSQGETMSALYRMATDEHRHISIKPNDLVIISA  467
CpyRNase      VNLDKKIFIDANEVSKYPDNEVLIVTTGSQGETMSALYRMATDEHKYIKIKPTDQIIISS  430
              ::     *:  .::      :: :: *****: *:.* *:* . *  : :   * :::*:

DraRNase      NPIPGNEDAVNLIVNRLYEIGVDVVYPPTYRVHASGHASQEELATILNLTRPKFFLPWHG  404
```

```
SpyRNase        SPIPGNTTSVNKLINTIQEAGVDVIHGKVNNIHTSGHGGQQEQKLMLSLIKPKYFMPVHG   393
BsuRNase        SPIPGNTISVSRTINQLYRAGAEVIHGPLNDIHTSGHGGQEEQKLMLRLIKPKFFMPIHG   391
SauRNase        SPIPGNTKSINRTINSLYKAGADVIHSKISNIHTSGHGSQGDQQLMLRLIKPKYFLPIHG   391
HpRNase         KAIPGNEASVSAVLNFLIKKEAKVAYQEFDNIHVSGHAAQEEQKLMLRLIKPKFFLPVHG   527
CpyRNase        KAIPGNETSVSTVLNYLLKSGASVAHQDFSEIHVSGHAAQEEQKLMLRLVKPKFFLPVHG   490
                . ****  ::.  :* : .  ..* :     :*.***..* :   :* * :**:*:* **


DraRNase        EPRHQINHAKLAQTLPRPPKRTLIAKNGDIVNLGPDEFRVSGTVAAGAVYVDGLGVGDVN   464
SpyRNase        EYRMQKIHAGLAMDIGIPKENIFIMENGDVLALTSDSARIAGHFNAQDIYVDGNGIGDIG   453
BsuRNase        EYRMQKMHVKLATDCGIPEENCFIMDNGEVLALKGDEASVAGKIPSGSVYIDGSGIGDIG   451
SauRNase        EYRMLKAHGETGVECGVEEDNVFIFDIGDVLALTHDSARKAGRIPSGNVLVDGSGIGDIG   451
HpRNase         EYNHVARHKQTAISCGVPEKNIYLMEDGDQVEVGPAFIKKVGTIKSGKSYVDNQSNLSID   587
CpyRNase        EYNHIVRHKETAIACGVDERNTYLMSDGDQIEVCQKYIKRLKTVKTGKVFIDNQINKQIS   550
                * .    *   .        .  : . *: : :          . :    :*.    .:.

DraRNase        DDVLLDRVNLSQEGLLILTAVLHPTPH-----VEVVARGFAR--PNRDLELQIRRVALEA   517
SpyRNase        AAVLRDRRDLSEDGVVLAVATVDFNTQMILAGPDILSRGFIYMRESGDLIRESQRVLFNA   513
BsuRNase        NIVLRDRRILSEEGLVIVVVSIDMDDFKISAGPDLISRGFVYMRESGDLINDAQELISNH   511
SauRNase        NVVIRDRKLLSEEGLVIVVVSIDFNTNKLLSGPDIISRGFVYMRESGQLIYDAQRKIKTD   511
HpRNase         TSIVQQREEVASAGVFAATIFVNKNKQALLESSQFSSLGLVGFKDEKHLIKEIQGGLEML   647
CpyRNase        DDVVIDRQKLAEAGVVTIISQIDKNAKTLIQN-RVISYGLVSERQSKNLSKEMEEVLLQF   609
                  :: :*  ::. *:.     :.           . : *:     . .*  : .

DraRNase        VEQGLR--EKKRLEDVRDDMYGAVRRFTRKATGRNPVLIPMIVD----------         559
SpyRNase        -IRIALKNKDASIQSVNGAIVNALRPFLYEKTEREPIIIPMVLTPDKH------         560
BsuRNase        -LQKVMERKTTQWSEIKNEITDTLAPFLYEKTKRRPMILPIIMEV---------         555
SauRNase        VISKLNQNKDIQWHQIKSSIIETLQPYLFEKTARKPMILPVIMKVNEQKESNNK         565
HpRNase         L-KSSNAEILNNPKKLEDHTRNFIRKALFKKFRKYPAIICHAHSF---------         691
CpyRNase        L-SNVKDELLHDQRALENQIRQVIRKHIFRKIKKYPTIVPVVYLM---------         653
                               :..     :     .   : * ::
```

Supplementary Figure 10

Side view (panel A) and top view (panel B) of the cartoon representations of the dimerization domain of RNase J based on the crystal structure (data available at PDB 7PCR). Red stick representations demonstrate the location of negatively charged amino acid residues E618, E654 and E663; blue stick representations show the positions of positively charged amino acid residues K649 and K660.

Panel A

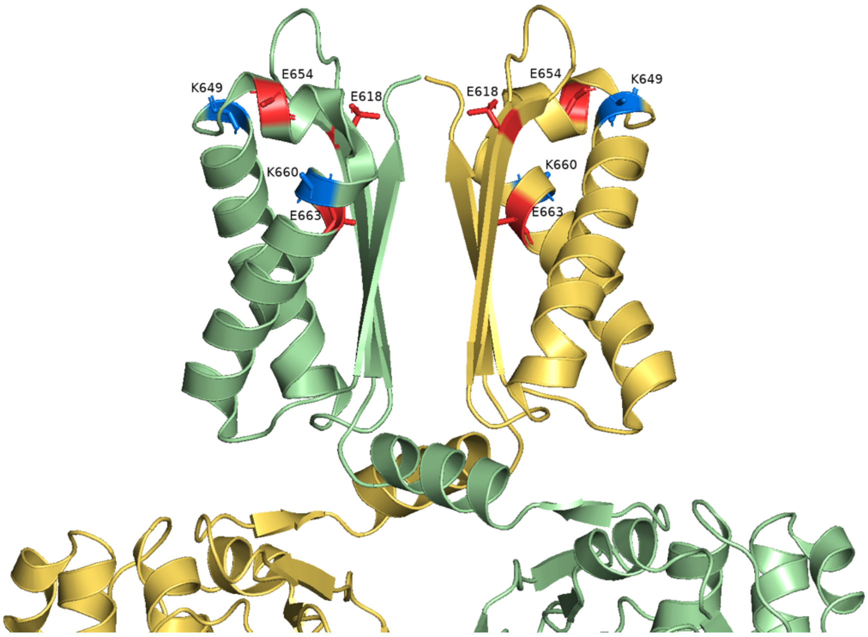

Panel B

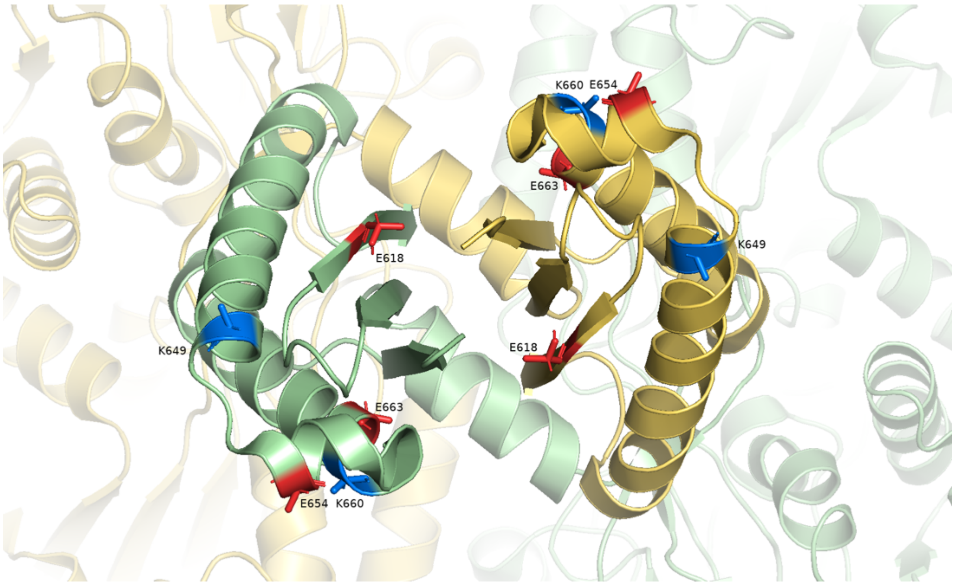

Supplementary Tables

Supplementary Table 1:

A) RNase J crystallographic data collection and refinement statistics.

The model and structure factors have been deposited in the PDB with accession code 7PCR.

	**RNase J core**
**Wavelength**	0.97778
**Resolution range**	56.03 - 2.75 (2.848 - 2.75)
**Space group**	I 41 2 2
**Unit cell**	158.49 158.49 214.26 90 90 90
**Total reflections**	128437
**Unique reflections**	35426 (3469)
**Multiplicity**	3.6 (3.6)
**Completeness (%)**	99.17 (99.06)
**Mean I/sigma(I)**	5.9 (1.3)
**Wilson B-factor**	75.00
**R-merge**	0.137 (0.80)
**R-meas**	0.160 (0.94)
**R-pim**	0.081 (0.486)
**CC1/2**	0.979 (0.453)
**Reflections used in refinement**	35419 (3467)
**Reflections used for R-free**	1805 (165)
**R-work**	0.2009 (0.3197)

**R-free**	0.2318 (0.3620)
**Number of non-hydrogen atoms**	3559
**macromolecules**	3530
**solvent**	29
**Protein residues**	450
**RMS(bonds)**	0.009
**RMS(angles)**	1.05
**Ramachandran favored (%)**	92.86
**Ramachandran allowed (%)**	6.47
**Ramachandran outliers (%)**	0.67
**Rotamer outliers (%)**	2.07
**Clashscore**	6.06
**Average B-factor**	72.57
**macromolecules**	72.62
**solvent**	67.25

Statistics for the highest-resolution shell are shown in parentheses.

B) RNase J cryo-EM data collection, refinement and validation statistics

The atomic coordinates and cryo-EM density map of RNase J have been deposited in the RCSB Protein Data Bank (PDB) with the accession code 8CGL and in the Electron Microscopy Data Bank (EMDB) with the accession code EMD-16647.

	RNase J
**Data collection and processing**	
Magnification	92000
Voltage (kV)	200
Electron exposure (e–/Å^2)	40.58
Defocus range (μm)	-3.0 − -1.2

Pixel size (Å)	1.106
Symmetry imposed	D2
Initial particle images (no.)	505965
Final particle images (no.)	34061
Map resolution (Å)<br>FSC threshold	4.19<br>0.143
Map resolution range (Å)	
**Refinement**	
Initial model used (PDB code)	7PCR
Model resolution (Å)<br>FSC threshold	4.2<br>0.143
Model resolution range (Å)	20-4.2
Map sharpening *B* factor (Å^2)	140
Model composition<br>Non-hydrogen atoms<br>Protein residues.<br>Ligands	 10912<br>2212
B factors (Å^2)<br>Protein.<br>Ligand	 156
R.m.s. deviations<br>Bond lengths (Å)<br>Bond angles (°)	 0.008<br>1.33
Validation<br>MolProbity score.<br>Clashscore.<br>Poor rotamers (%)	 1.8<br>6.2<br>0
Ramachandran plot<br>Favored (%)<br>Allowed (%)<br>Disallowed (%).	 93<br>7<br>0

Supplementary Table 2. Estimation of the proportions of the secondary structure elements of wild type RNase J and variant K649A deduced from their circular dichroism spectra.

Secondary structure	RNase J WT (%)	RNase J K649A (%)
Alpha-helix	4.4	5.2
Parallel beta-sheet	0	0
Antiparallel beta-sheet	37.5	36.9
Turn	14.4	14.5
Others	43.5	43.4

Supplementary References

Boneca, I. G., Ecobichon, C., Chaput, C., Mathieu, A., Guadagnini, S., Prévost, M.-C., Colland, F., Labigne, A., & de Reuse, H. (2008). Development of inducible systems to engineer conditional mutants of essential genes of *Helicobacter pylori*. *Applied and Environmental Microbiology*, *74*(7), 2095–2102. https://doi.org/10.1128/AEM.01348-07

El Mortaji, L., Aubert, S., Galtier, E., Schmitt, C., Anger, K., Redko, Y., Quentin, Y., & De Reuse, H. (2018). The sole DEAD-box RNA helicase of the gastric pathogen *Helicobacter pylori* is essential for colonization. *MBio*, *9*(2), e02071-17. https://doi.org/10.1128/mBio.02071-17

Farnbacher, M., Jahns, T., Willrodt, D., Daniel, R., Haas, R., Goesmann, A., Kurtz, S., & Rieder, G. (2010). Sequencing, annotation, and comparative genome analysis of the gerbil-adapted *Helicobacter pylori* strain B8. *BMC Genomics*, *11*(1), 335. https://doi.org/10.1186/1471-2164-11-335

Karimova, G., Pidoux, J., Ullmann, A., & Ladant, D. (1998). A bacterial two-hybrid system based on a reconstituted signal transduction pathway. *Proceedings of the National Academy of Sciences of the United States of America*, *95*(10), 5752–5756. https://doi.org/10.1073/pnas.95.10.5752

McClain, M. S., Shaffer, C. L., Israel, D. A., Peek, R. M., & Cover, T. L. (2009). Genome sequence analysis of *Helicobacter pylori* strains associated with gastric ulceration and gastric cancer. *BMC Genomics*, *10*(1), 3. https://doi.org/10.1186/1471-2164-10-3

Miroux, B., & Walker, J. E. (1996). Over-production of proteins in *Escherichia coli*: Mutant hosts that allow synthesis of some membrane proteins and globular proteins at high levels. In *Journal of Molecular Biology* (Vol. 260, Issue 3, pp. 289–298). Academic Press. https://doi.org/10.1006/jmbi.1996.0399

Munier, H., Gilles, A.-M, Glaser, P., Krin, E., Danchin, A., Sarfati, R., & Bârzu, O. (1991). Isolation and characterization of catalytic and calmodulin-binding domains of *Bordetella pertussis* adenylate cyclase. *European Journal of Biochemistry*, *196*(2), 469–474. https://doi.org/10.1111/j.1432-1033.1991.tb15838.x

Tejada-Arranz, A., Galtier, E., El Mortaji, L., Turlin, E., Ershov, D., & De Reuse, H. (2020). The RNase J-Based RNA Degradosome Is Compartmentalized in the Gastric Pathogen *Helicobacter pylori*. *MBio*, *11*(5), e01173-20.

https://doi.org/10.1128/mBio.01173-20.
