# Supplementary material for: Acetylation regulates the oligomerization state and activity of RNase J, the Helicobacter pylori major ribonuclease
Source: Nat Commun. 2023 Dec 6;14:8072. doi: 10.1038/s41467-023-43825-8 (PMC10700544; doi:10.1038/s41467-023-43825-8)
Supplement: Supplementary file 2 — Description of Additional Supplementary Files [file 41467_2023_43825_MOESM2_ESM.docx]

**Description of Additional Supplementary Files**

File Name: Supplementary Data 1

Description: Mass spectrometry data of the raw intensity of the acetylated RNase J peptides, the peptides used for normalization across conditions and the normalized data.

File Name: Supplementary Data 2

Description: List of strains and plasmids used in this study.

File Name: Supplementary Data 3

Description: List of oligonucleotides used in this study.
